# Intratumoral generation of photothermal gold nanoparticles through a vectorized biomineralization of ionic gold

**DOI:** 10.1038/s41467-020-17595-6

**Published:** 2020-09-10

**Authors:** Aaron S. Schwartz-Duval, Christian J. Konopka, Parikshit Moitra, Enrique A. Daza, Indrajit Srivastava, Elyse V. Johnson, Taylor L. Kampert, Stanley Fayn, Anand Haran, Lawrence W. Dobrucki, Dipanjan Pan

**Affiliations:** 1 Department of Bioengineering, University of Illinois, Urbana-Champaign, Urbana, IL 61801 USA; 2 Beckman Institute, University of Illinois, Urbana-Champaign, Urbana, IL 61801 USA; 3 Biomedical Research Center, Carle Foundation Hospital, Urbana, IL USA; 4 Department of Diagnostic Radiology and Nuclear Medicine, University of Maryland Baltimore School of Medicine, Baltimore, MD 21201 USA; 5 Department of Pediatrics, Center for Blood Oxygen Transport and Hemostasis, University of Maryland Baltimore School of Medicine, Health Sciences Research Facility III, 670 W Baltimore St., Baltimore, MD 21201 USA; 6 Department of Chemical, Biochemical and Environmental Engineering, University of Maryland Baltimore County, Interdisciplinary Health Sciences Facility, 1000 Hilltop Circle Baltimore, Baltimore, MD 21250 USA; 7 Cytoviva inc., Auburn, 36830 USA

**Keywords:** Nanoparticles, Nanotechnology in cancer, Nanoparticles

## Abstract

Various cancer cells have been demonstrated to have the capacity to form plasmonic gold nanoparticles when chloroauric acid is introduced to their cellular microenvironment. But their biomedical applications are limited, particularly considering the millimolar concentrations and longer incubation period of ionic gold. Here, we describe a simplistic method of intracellular biomineralization to produce plasmonic gold nanoparticles at micromolar concentrations within 30 min of application utilizing polyethylene glycol as delivery vector for ionic gold. We have characterized this process for intracellular gold nanoparticle formation, which progressively accumulates proteins as the ionic gold clusters migrate to the nucleus. This nano-vectorized application of ionic gold emphasizes its potential biomedical opportunities while reducing the quantity of ionic gold and required incubation time. To demonstrate its biomedical potential, we further induce in-situ biosynthesis of gold nanoparticles within MCF7 tumor mouse xenografts which is followed by its photothermal remediation.

## Abstract (web summary)

Intracellular generation of gold nanoparticles has drawn attention but toxic effects have limited potential applications. Here, the authors report on the delivery of ionic gold with PEG resulting in faster synthesis and reduced toxicity due to lower concentrations of ionic gold required and explore potential applications.

## Introduction

Gold nanoparticles have been extensively utilized for biological applications due to their simplistic and tunable chemistry which provides the ability to control and modify size, morphology, and surface functionality; combined with their morphology-dependent^1–4^. Typically, generating gold nanoparticles require, at the minimum, chloroauric acid as a precursor material and a reducing agent for catalyzing the reductive nucleation. Variations in the reducing agent molecule as well as reaction conditions (e.g. pH, temperature, incubation time) will result in different morphologies, sizes, and functionalities of the resulting nanoparticles^5–8^. For biomedical applications, these variations in morphology, size, and functionality are directly correlated to their cellular internalization^9–11^, biodistribution, biological half-life^11–13^, renal secretion^11–15^, and plasmon optical properties^1–8^. Because of their plasmon-optical properties, gold nanoparticles are inherently theranostic, providing imaging contrast through near infrared (NIR)^16,17,15,16^, fluorescence^18,19^, X-Ray^20,21^, photoacoustic^22–24^, and Raman enhancement^25,26^. This plasmonic optical property therefore allows gold nanoparticles to act as a vehicle to enhance ablative therapies both for photothermal^27,28^, and X-ray radiation^29–31^.

On the other hand, the bio-inertness of gold nanoparticles combined with the diverse range of applicable reductant molecules has allowed researchers to develop biomimetic gold nanoparticles using biomolecules as scaffolds for its reduction^32–35^. Therefore, this approach requires no additional steps for bioconjugation since the reducing biomolecules remain attached as capping agents to the formed nanoparticles. Further to this, if cellular machinery are used to generate gold nanoparticles, the resulting plasmon-optical properties could act as a reporter of the affecting cell phenotype. This capability to generate gold nanoparticles via cellular biomolecules has already been demonstrated previously in mammalian cells^36–47^. The precise mechanism for this cellular bioreduction of gold nanoparticles is not fully understood, however, these treatments have been known to negatively impact cell health through cellular responses via shock and stress-related proteins^42^, decrease in viability^43,44^, and even cell fixation^44^. These previous works, wherein ionic gold was applied diffusely to generate gold nanoparticles via cellular mechanisms, could not be directly applied for cellular diagnostics or therapeutics, as the process is so deleterious to cell health and not cell-specific. However, this process of cellular bioreduction forming gold nanoparticles has been marketed as a means for generating biomimetic and biofunctional gold nanoparticles, which could potentially be applied to different physiological systems. One such recent work by A.V. Singh et al. demonstrated the photothermal effects from intracellular gold, reduced in vitro through the addition of prefabricated gold nanoparticle seeds^36^. This work demonstrated that the process of biomineralization could easily be modified through suitable tuning in the recipe of precursor materials.

In this work, we hypothesize that if ionic gold are deployed to cells in discrete nanoscaled packets, this can largely reduce the concentration of gold necessary for the reduction, as well as decrease the time frame and overall impact on cell health, thereby broadening its use for diagnostic and therapeutic purposes. Accordingly, a simplistic delivery vehicle has been demonstrated herein for deploying ionic gold, which indicate intracellular nanoparticle formation within a time span of ~30 min, much less than the previous literature reports, with concentrations lower than previous applications, in complete cell media. Additionally, we have identified proteins and their respective subcellular locations involved in the cellular bioreduction of gold nanoparticles providing larger insight into this potential reduction pathway. The process of sequential and progressive reduction of gold nanoparticle has been shown to occur initially via secreted proteins in the extracellular space and finally within the nucleus for terminal reduction. Because this approach has minimal impact on protein expression/integrity and some of the bound proteins are nucleotide binding, there is a strong potential of the developed gold nanoparticles to be used for theranostic application to provide photothermal ablation directly to the nucleotides. Thus, we demonstrate a theranostic application through a MCF-7 cancer xenograft model, wherein fluorescent and photothermally active gold nanoparticles are formed intracellularly to remediate cancer xenografts.

## Results

### Vehicle design and characterization for ionic gold delivery

**Fig. 1:**
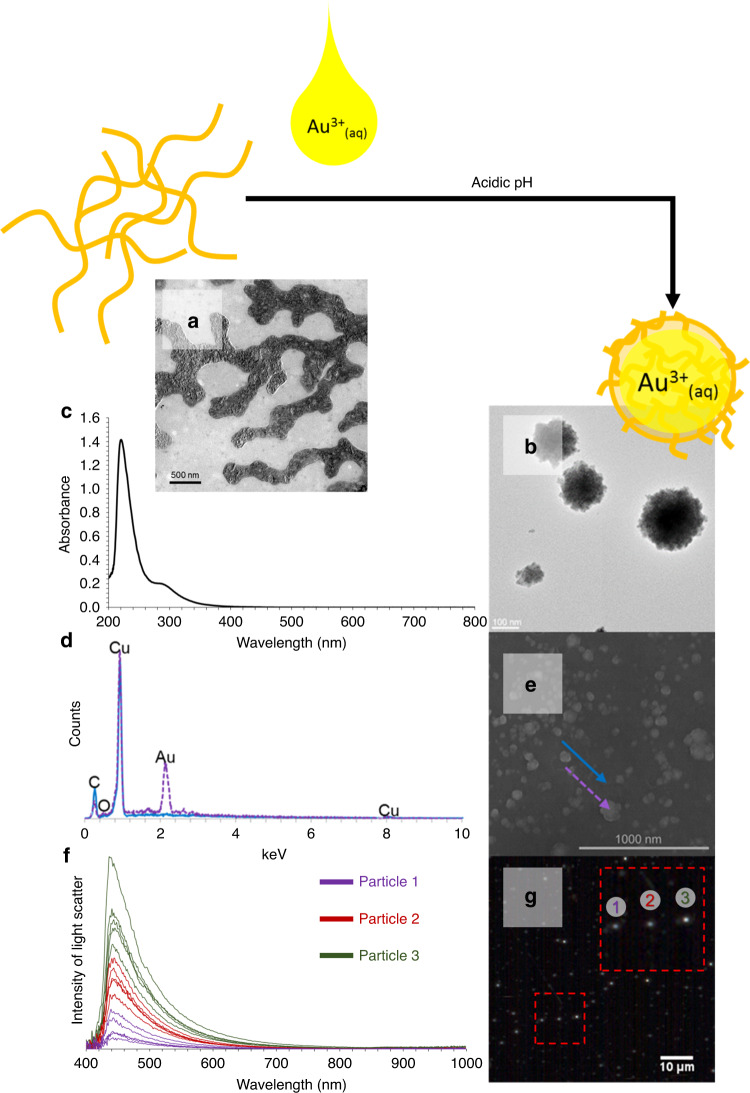
Characterization of ionic Au-PEG delivery vehicle. Representative transmission electron microscopy (TEM) images of (**a**) PEG and (**b**) ionic Au–PEG clusters. **c** UV–vis absorbance spectrum of ionic Au–PEG clusters (200–800 nm). **d** Electron dispersion spectrum of ionic–Au–PEG clusters (on copper tape) captured from (**e**) scanning electron microscopy-energy dispersive spectra (SEM-EDS). Purple spectra correspond to signal obtained from the clusters, where the blue spectra corresponds to the background. **f** Spectral intensity of light scatter obtained through enhanced dark field hyperspectral imaging of discrete Au–PEG clusters in solution (*n* = 3 particles, with 5 spectra from each). **g** Enhanced darkfield hyperspectral image with 2X digital magnification where the red box highlights the particles those represent the spectra as shown in (**f**).

For intracellular gold nanoparticle formation, all of the constituents (sans chloroauric acid) are innately present in cellular systems. This intracellular gold nanoparticle formation has been demonstrated previously in mammalian cells, however, only through bulk diffusive treatments, lasting more than 24 h, and typically those are in solutions which contain no cell nutrients (such as DPBS)^35–47^. These treatment scenarios are non-ideal for minimizing the impact on cellular health. To minimize the caustic properties of chloroauric acid, we aim to deploy ionic gold salts in discrete nano-scaled clusters. It was hypothesized that if we are successful in deploying ionic gold in discrete nanoscaled clusters, it could potentially allow us to reduce the overall dosage, required for intracellular gold nanoparticle (GNP) formation, and in turn, reduce the impact on cellular physiology. In order to successfully enable intracellular reduction of ionic gold by the action of cellular biomolecules, it is necessary that the ionic gold delivery vehicle: (a) is made of material that will not spontaneously reduce ionic gold, (b) will contain the ionic gold in discrete nanoscale packets, (c) will have the capacity to interact with the cells, and (d) that cellular biomolecules will have access to the gold ions for nanoparticle formation. While chloroauric acid can be readily reduced by a large varieties of functional groups, it is not readily reduced by ether groups, carbon–carbon single bonds, nor under acidic pH conditions. With this knowledge in hand, hydroxyl terminated polyethylene glycol (PEG) was used as the ionic Au^3+^ delivery vehicle. The rationale behind this was that PEG is known to cluster with ionic gold in acidic pH solutions^6^, and will not cause spontaneous gold nanoparticle (GNP) formation. We do not suspect that PEG would induce spontaneous GNP formation as polyol-based Au/Ag nanoparticle synthesis, where PEG could function as a reductant, typically require much higher temperatures (160 °C) than utilized for cell culture (37 °C)^48^. In a typical process for the formation of ionic gold delivery vehicle, HAuCl_4_ (14 µmol) and PEG (5 µmol) were co-clustered in a sealed glass vial with 2 ml of carbon filtered deionized water (pH = 4). We observed that PEG molecules formed clusters in acidic conditions when compared to neutral conditions. In addition, positively charged gold ions were seen to preferentially localize within these clusters (Fig. 1a, b). This mixture of ionic gold and PEG was then incubated in a water bath kept at 37 °C for 30 min to ensure thermal equilibrium achieved before dilution for analysis and/or cell treatment. A UV–Vis electromagnetic spectra confirmed that the gold in these Au-PEG clusters was ionic and not reduced in nature (Fig. 1c) due to the lack of absorbance peaks that are characteristic of plasmon formation. This spectrum revealed an absorbance spectra typical of ionic gold (Supplementary Fig. 1a), with no observable peak in the 500–700 nm range that is characteristic of the surface plasmonic resonance of reduced gold^1–4^. Additionally, no visible color change or precipitant formation was observed if the solution was left undisturbed on the benchtop (RT) for 30 days (all treatments and samples used for analysis were made fresh). The discrete Au-PEG clusters were 288 ± 46 nm in diameter, as quantified by transmission electron microscopy (TEM) (Fig. 1b). From scanning electron microscopy–energy dispersive spectrum (SEM–EDS), we confirmed that the Au-PEG clusters contain gold (Fig. 1d, e) that is localized within the PEG clusters rather than dispersed within the solution. The dark contrast in TEM afforded by these clusters (Fig. 1b), as well as light scatter from dark field hyperspectral imaging of the clusters in solution (Fig. 1f, g) further supports these observations that the ionic gold is localized within the PEG clusters. The light scatter afforded through enhanced dark-field hyperspectral imaging for the non-reduced ionic gold particles confirmed the lack of surface plasmon resonance peaks that would correspond to the reduced gold. The light scattering peak at 420 nm is an artifact of the Vis–NIR liquid light guide which transmits light beginning at 420 nm. Thus, using these spectral and microscopic techniques, we have successfully characterized our delivery vehicles as containing ionic gold in discrete nanoscaled packets (without auto-reduction).

### Forming gold nanoparticles via cellular mechanisms

**Fig. 2:**
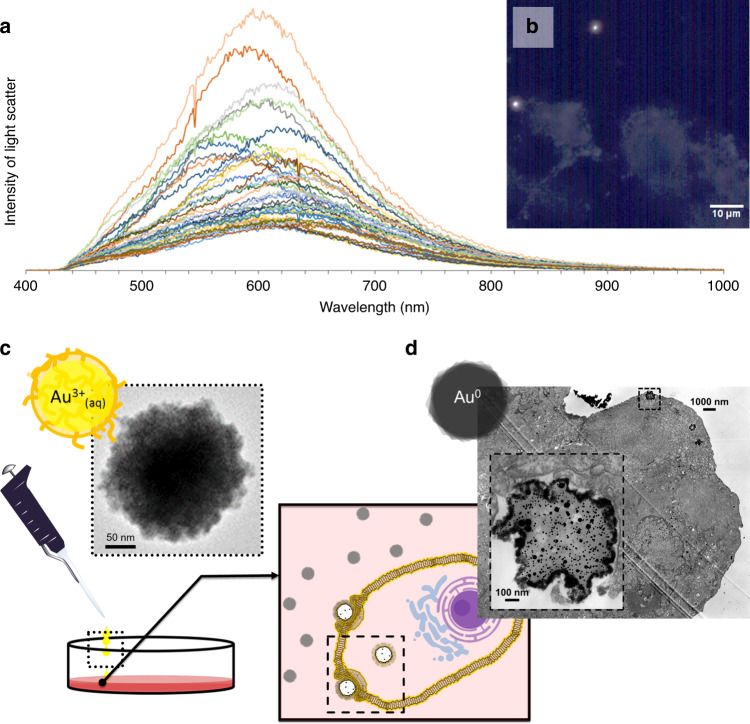
Confirmation of in vitro reduction occurring en route. **a** Spectral intensity of light scatter from MCF-7 cells treated with Au–PEG clusters (*n* = 44 spectra), as distinct from untreated MCF-7 cells obtained through hyperspectral enhanced dark field imaging. **b** Corresponding enhanced hyperspectral darkfield image mapping of nanoparticles generated through Au–PEG treatment to MCF-7 cells with negative filtering with untreated MCF-7 cells (Supplemental Fig. 1e) to ensure no false-positive nanoparticle mapping. Scheme (**c**, **d**) with representative TEM images describing the reduction of ionic Au–PEG clusters (**c**), upon their interaction with cells (**d**).

**Fig. 3:**
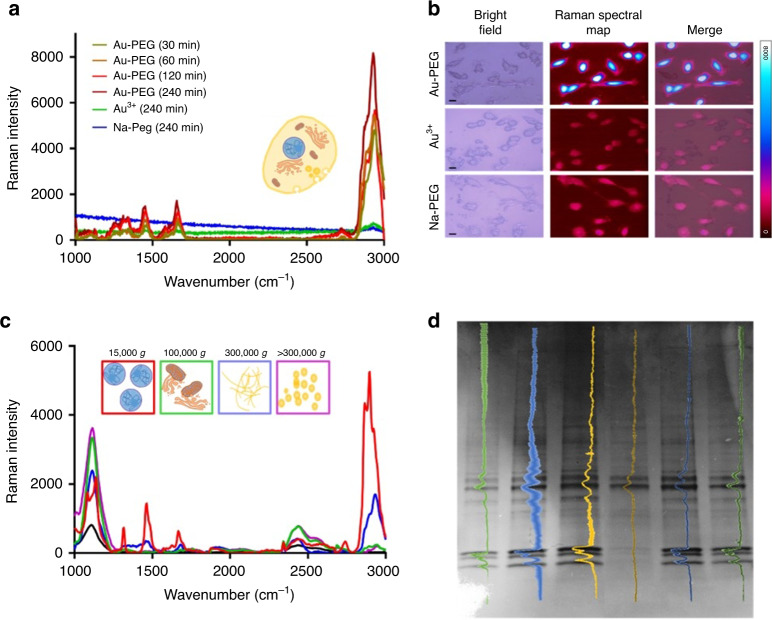
Characterization of intracellular reduction of ionic gold. Raman spectra (**a**) of cells treated with either ionic Au–PEG clusters after 240 min (burgundy), 120 min (red), 60 min (orange), 30 min (yellow), Au^3+^ (green), or Na–PEG (blue). Raman mapping (of 2800–3000 peak intensity) with the corresponding bright field and merged images for 240 min treatments of Au–PEG, Au^3+^ and Na–PEG (**b**). Scale bars for (**b**) are 20 µm. **c** Raman spectra of Au–PEG-treated MCF-7 cellular fractions separated using differential centrifugation including the nucleus and large organelles, 15,000 *g* (red); mitochondria, lysosomes and other medium sized organelles, 100,000 *g* (green); membrane fragments, 300,000 *g* (blue); and highly soluble/cytosolic molecules (violet), >300,000 *g*; and the glass slide without cell fractions (black). **d** SDS-PAGE stained with Coomassie Blue with intensity plots overlaid of protein from the nuclear fraction of MCF-7 cells without treatment (green), treated with Na–PEG (blue), or Au–PEG (yellow) with standard SDS-PAGE conditions (lighter-shaded colors, left three lanes), or without β-mercaptoethanol (darker-shaded colors, right three lanes).

Ionic Au-PEG clusters, prepared with 0.24 mM Au^3+^, were capable of enabling a cellular reduction within 4 h of treatment in human breast cancer cell cultures (MCF-7) in complete cell media (containing 10% FBS, with 1x penicillin and streptomycin). Previous applications for generating gold nanoparticles intracellularly, typically apply 1 mM of ionic gold over time periods >24 h^36–47^. However, the amount of formed plasmonic gold nanoparticles were negligible or considerably reduced when the ionic gold was applied diffusively in the previous literature reports either at lower concentrations (0.1 mM^43,44^, 0.25 mM^42^) or for shorter time periods (12 h^42^, 15 h^45^). From our application, we were able to confirm the transition of ionic to plasmonic gold through the spectral library obtained from the enhanced dark-field hyperspectral imaging of Au-PEG clusters reduced through the interactions with MCF-7 cells (Fig. 2a, b). Hyperspectral imaging technique was used to investigate the intracellular bio-reduction process of ionic gold. It was found that although the treated precursor concentration was quite low than the previously reported ones, characteristic surface plasmon bands for Au(0) could only be observed after the bio-reduction process (Fig. 2a) in contrast the absence of plasmonic peaks for Au-PEG clusters (Fig. 1f). This further confirms that the bio-reduction process led to the formation of AuNPs with the oxidation state of Au(0) from the +3 state at Au-PEG ionic clusters. To confirm that these plasmonic nanoparticles were internal and not simply on the cell surface, TEM imaging of MCF-7 cells treated with Au-PEG clusters was performed (Fig. 2c and Supplementary Fig. 1d). An electron density focusing in formed particles with a dark, continuous, irregularly shaped border along the periphery was observed during this experiment (Fig. 2c). Further to confirm that gold nanoparticle formation is occurring en route to cellular internalization and not by undesired reactions with either the culture plate coating or the culture media, we undertook several control experiments. One such possibility is the presence of high number of primary amines on the adherent culture plate surface (i.e. poly-l-lysine coating) may induce reduction of ionic gold within the solution of the culture plate. But no reductive or structural changes were observed in this case even after incubating the ionic Au-PEG clusters for 24 h at 37 °C on standard culture plates with poly-l-lysine coating (Supplementary Fig. 1b). Bovine serum albumin (BSA), a protein nutrient source within the culture media, is capable of reducing gold nanoparticle with the aid of mild to strong reductants, heating or pH manipulation (outside of physiological norms)^48,49^. We confirmed that BSA was not the source of gold biomineralization in our system, as the fresh culture media (containing BSA) was not able to reduce the ionic gold clusters (24 h, 37 °C) as confirmed through TEM images of this treatment solution (Supplementary Fig. 1c). For BSA-based biomineralization procedures, it is necessary to include strong/mild reductants or basic pH manipulation^49,50^. Contrary to these, neither strong/mild reductants, pH manipulation, or heating (beyond physiological norms) are present within the protocol of our system. Further the final pH of the cell surrounding was tested while HAuCl_4_ was added to the cell media for a final concentration of 1 mM, more than four times the concentration that was used in the current study. We observed that the change in pH was <0.1 suggesting the claim of benign environment for the biomineralization of gold nanoparticles. Thus, to confirm that our gold nanoparticle formation is enhanced directly as a result of our delivery vehicle, Raman micrographs of cells treated with Au-PEG clusters, chloroauric acid (Au^3+^) (lacking PEG), and a negative control containing PEG and sodium salt (Na^+^) in equal molar concentration of the analyte (Au^3+^) were acquired. It was observed that the Raman intensity for the cells treated with Au-PEG were markedly enhanced (8×) compared to cells treated with Na-PEG or just chloroauric acid only after four hour of incubation period (Fig. 3a, b). Additionally, this Au-PEG treatment begins to provide plasmonic enhancement as early as 30 min after treatment (Fig. 3a). This enhancement can only arise from species within a range of <100 nm. This plasmonic enhancement, only found from the cells treated with Au-PEG, confirmed the essential role of discretization of the Au^3+^ cations within a delivery vehicle for plasmonic nanoparticle formation at the applied concentrations and time frame.

### Investigation on participating biomolecules

In order to utilize intracellularly formed GNPs directly for the biomedical applications (either diagnostic, therapeutic or a combination of both), knowledge of their formation process induced by the cellular biomolecules must be obtained. Elucidating the formation process and knowledge on the participating biomolecules for the formation of these gold nanosystems would provide valuable information relating to the possible pathological pathways with which this process could potentially be suitable for diagnostic/therapeutic applications. The enhanced Raman spectral maps afforded by ionic Au-PEG treatments to MCF-7 cells (Fig. 3a, b) provided important chemical information regarding the biomolecules proximal to the intracellularly formed nanoparticles. The biomolecules, proximal to the intracellularly formed nanoparticles, may be bound to or involved in process of their formation. The enhanced Raman spectra contain characteristic peaks arising from lipid (1175–1385, 1446–1477, and 2849–2969 cm^−1^), and proteins (1590–1650 cm^−1^)^51^. Additionally, lack of enhanced characteristic phosphate peaks at 2380–2450 cm^−1^ in the enhanced Raman spectra allowed us to confirm that phosphates from the sodium phosphate buffer and/or the nucleic acids are not participating in this gold reduction (Fig. 3a)^51^. However, the presence of amide, CH_3_, and CH_2_ peaks do not provide enough information on their own to be considered viable as a diagnostic tool. In addition, this information does not prove precisely what physiologic processes are occurring within our system. To more precisely determine the biomolecules engaged in this intracellular reduction pathway, and which portions of the cell they originate from, cellular fractions of cells treated with the ionic gold nanoclusters were separated by density (through differential centrifugation) and the presence of plasmonic gold was subsequently investigated through acquisition of Raman spectra (SERS) (Fig. 3c). To generate these cellular fractions, mechanical lysis by tip sonication (30 s at 1 A) of MCF-7 cells treated with the ionic Au-PEG or untreated cells was performed^52^. Then the fractions were separated by density via differential centrifugation into fractions typically associated with organelles of different sizes, i.e. (i) nuclei and large organelles, pellet from 15,000 × *g* for 5 min; (ii) mitochondria, lysosomes, peroxisomes, medium organelles, pellet from 100,000 × *g* for 60 min; (iii) cellular membrane fragments, pellet from 300,000 × *g* for 120 min; and (iv) highly soluble peptides/cytosolic molecules, supernatant from 300,000×g for 120 min)^53^. For these fractions, corresponding Raman spectra were collected from cells either treated and or not treated with Au-PEG (Fig. 3c and Supplementary Fig. 2). Significant enhancement of the Raman spectra was observed for the fraction containing nuclei and the fraction containing cell membrane fragments from the cells treated with Au-PEG. Both the nuclear fraction and cellular membrane fragment fraction contained noticeable amide peaks at 1590–1650 cm^−1^ ^51^. Due to the prominent amide peak, we were led to believe that these proteins were playing a significant role in this intracellular reduction process. The enhanced Raman spectra found in nuclear fraction, which implies localization of Au-PEG to the nucleus, does not necessarily imply internalization of the particles to the nucleus. Since the Raman microscope imaging is 2D, we cannot make this assertion from the cell imaging either. However, since there is a distribution of nanoparticle sizes, from sub 5 nm to larger, it could be inferred that some smaller particles may pass through the nuclear pores (~6 nm) to get internalized.

The notion, that proteins are involved in cellular biomineralization, has been explored by Singh et al.^42^, however, their system is resultant from diffuse applications of Au^3+^ so we should not infer that these systems/processes are identical. To determine if proteins are involved in our intracellular gold nanoparticle formation, a Coomassie Blue stained, sodium dodecyl sulfate–polyacrylamide gel electrophoresis (SDS–PAGE) was used to separate proteins found in the fractions from cells treated with Au-PEG, Na-PEG, or untreated (Fig. 3d and Supplementary Fig. 3a–d). We hypothesized that from Au-PEG and Na-PEG treatments, protein band intensity may be affected; and from the Au-PEG treatment, nanoparticle-bound proteins could be slowed in their electrophoretic migration through the gel due to the additional mass of gold. From this gel, we found no discernable difference between specific bands present nor their relative expression between treatments (Fig. 3d and Supplementary Fig. 3a–d). The information provided by this gel posed two possible major implications—(1) none of our treatments were affecting protein expression/integrity, and, all proteins which were running through the gel in detectable quantities did not adhere to the gold nanoparticles, and/or (2) proteins were not involved in this process. Another possible explanation, which we explored, was that one of the reagents used for preparing the protein for SDS–PAGE separation may have possibly interfered with the protein–gold interactions, effectively freeing the involved proteins from the nanoparticles. The primary candidate considered for this interference was β-mercaptoethanol (βME), as it is known to function as a highly efficient capping/quenching agent for gold nanoparticle syntheses capable of breaking Au–S bonds between nanoparticles and protein^54^. To determine whether βME was responsible for this freeing of Au-bound proteins, we were prompted to run an additional gel comparing protein migration from the fractions of cells either treated with Au-PEG, Na-PEG, or no treatment in duplicate—half would receive βME and the other half would not (Fig. 3d and Supplementary Fig. 3a–d). From these protein gels, we found distinct band intensity differential in the 10,000 × *g* (nuclei and large organelle) protein fraction between the well with Au-PEG βME (−) and all other wells of the same gel (Fig. 3d and Supplementary Fig. 3a–d). The identical protein bands observed in control, Na-PEG, and Au-PEG of βME containing lanes indicated that neither the Na-PEG nor the Au-PEG treatment affected the protein expression/integrity of proteins found within the nuclear fraction. Comparing this observation with the lanes of the gel that were lacking βME, we found that changes in protein band intensity were observed only in the Au-PEG lane. These changes in protein band intensity, which exclusively decreases in intensity, were only observed in the Au-PEG βME lacking lane. This indicated that several proteins within that layer are attached to the formed plasmonic gold nanoparticles and that these proteins have maintained integrity. Having observed protein impedance within the nuclear fraction of the ionic Au-PEG treated cells, we aimed to identify these proteins through LC–MS protein fingerprinting.

**Fig. 4:**
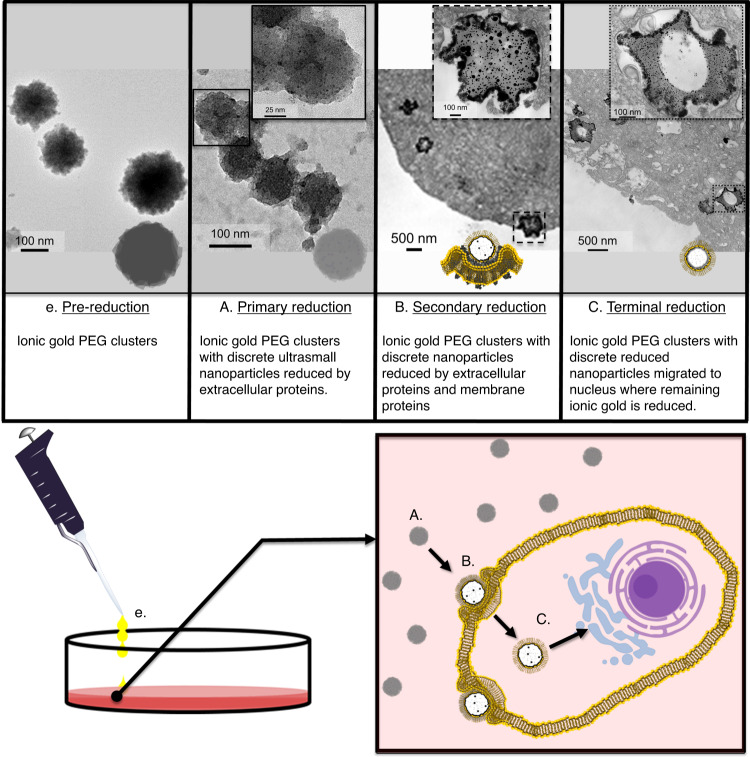
Scheme of progressive reduction process. Pictorial diagram for the intracellular formation of gold nanoparticles via Au–PEG clusters throughout their migration to the nucleus with TEM images of respective reduction phases.

In order to identify proteins involved in intracellular gold nanoparticle formation through mass spectroscopy, we separated involved proteins from those not involved through gel electrophoresis. From our previous Coomassie-stained gel experiments, we found that involved proteins would remain bound to the gold nanoparticles preventing their electrophoretic migration through the gel. To separate these gold bound proteins from the non-involved proteins, we would run the nuclear fraction of ionic Au-PEG treated cells without βME for an excess of time (>3 h), at which point the largest ladder protein would have run off the gel (250 kDa) and we would then separate the top portion of the gel containing the wells (1.5 cm) from the bulk (Supplementary Fig. 4). The proteins/gold particles, located within either of these gel portions, were then extracted using a diffusion-mediated passive elution method^55–57^. These extracted proteins were then briefly trypsinized to fragment the proteins present from extractions and centrifuged at 6000 × *g* to pellet any gold nanoparticles out of the solution. The supernatant containing soluble protein fragments was then analyzed via LC–MS with MASCOT analysis (Matrixscience, Boston, MA). We found almost 2239 different protein fragments within the top portion of the gel, which contained proteins that were most impeded through the gel. These protein fragments identified within the top portion of the gel, including only those with a protein score >21 (*α* < 0.05) and uniquely found in the top of the gel (excluding large slow moving proteins found in the bulk portion), accounted for 16 different proteins (Supplementary Tables 1, 2). These proteins are located, as annotated on UniProt.org through COMPARTMENTS^58^, include subcellular regions ranging from extracellular to the nucleus (Supplementary Table 1). These proteins of interest are involved in 287 different molecular processes as identified through phylogenetic-based propagation of functional gene ontology (GO) annotations (Supplementary Table 2)^59^. Of these molecular processes, the most conserved GO term processes are related to binding (Table 1). Eleven of the 16 identified proteins are involved in binding processes, eight of those in protein binding, five in metal/calcium/cationic binding, three in ATP/nucleic acid binding, two in antigen binding, two which directly bind with each other, and two which bind actin filament (Table 1). These 11 proteins, involved in molecular binding and the intracellular reduction of gold nanoparticles, are characteristically located in regions ranging from extracellular to the nucleus^58^. Since our extraction and isolation of these gold-bound proteins was from the nuclear fraction and many of these proteins are not typically located in the nucleus, this suggests that progressive intracellular reduction may occur and continue sequentially, accumulating a protein corona from multiple regions of the cell (Fig. 4). This proposed migratory path with sequential reduction towards the nucleus initiates as the ionic gold delivery vehicle interacts with the secreted proteins (Fig. 4a). To simulate this primary reduction process, which occurs extracellularly, we admixed ionic Au-PEG clusters with spent cell media (cells have been incubated with media for ~24 h). Through TEM imaging we were able to find electron density focusing within the PEG clusters potentially indicating nanoparticle formation (Fig. 4a). This electron density focusing is not observed when ionic Au-PEG clusters are admixed with fresh cell media (Supplementary Fig. 1b), indicating that this electron density focusing is resultant of cellular biomolecule secretions. The proteins involved in this primary reduction include those involved in antigen binding/phagocytosis (IGLL5, IGHA1), metabolic/catalytic activity (ZG16B, AMY2B, GBE1), cation binding (AMY2B and GBE1), and steroid binding (SCGB1D2)^59^. Two of these proteins involved in primary reduction are also located in multiple cellular regions (GBE1 and IGHA1) posing as potential migration enablers to the other cellular regions^58^. In addition, their localization to the extracellular regions, GBE1 and IGHA1 are also located within the cytoplasm and plasma membrane regions, respectively^58^. This potential for mitigating migration is especially notable for IGHA1, as this protein is both located in the plasma membrane and involved in receptor-mediated phagocytosis. The proteins GBE1 and IGHA1 could act as enablers for migration of the ionic Au-PEG clusters from the primary to secondary reduction phase, wherein the clusters interact with the plasma membrane (Fig. 4b). Through cellular TEM images of our ionic gold PEG clusters, we were able to see additional electron density focusing compared with that found via interactions with cell secretions as they interact with the protein-rich membrane (Fig. 4b). The additional electron density focusing shows nanoparticles within the clusters as more polydisperse than from the primary reduction with the addition of a dark electron-dense border contiguous with the plasma membrane. Additionally, from enhanced Raman signals found in the membrane of whole cells (Fig. 3a, b) and in membrane fractions (Fig. 3c), we concluded that plasmonic nanoparticles exist in the plasma membrane. This secondary reduction, catalyzed by the protein-rich membrane, may be initiated by IGHA1 as it found extracellularly, within the plasma membrane, and involved in receptor-mediated endocytosis. As this endocytosis is initiated, the ionic Au-PEG cluster is proximal to the plasma membrane, this proximity subjects the clusters to higher concentrations of cellular proteins/biomolecules, which are either secreted or located within the plasma membrane providing an environment more conducive to nanoparticle formation. The proteins involved in this secondary reduction within the plasma membrane are involved in cation binding (IGHA1, CDH23), antigen binding (IGHA1), kinase regulation/binding (SRCN1, ITGB1BP1), protein transport (ITGB1BP1), receptor clustering (ITGB1BP1), and endocytosis (IGHA1)^59^. In addition to IGHA1, SRCN1, and ITGB1BP1 are also found in multiple cellular regions as potential migratory enablers. Moreover being located within the plasma membrane, SRCN1 and ITGB1BP1 are both located within the cytoskeleton and ITGB1BP1 is located within the nucleus^58^. Once internalization of the ionic Au-PEG clusters is complete, we observed through TEM that the electron density at the center of these clusters diminishes (Fig. 4c). In addition to the hollowing of the Au-PEG clusters, the electron-dense border is less defined compared to the secondary reduction phase (during interaction with the plasma membrane). A possible explanation for this reduction in electron density, is that, as the Au-PEG clusters interact with the plasma membrane, some portion of the gold may remain within the plasma membrane after the Au-PEG clusters are internalized. It is worthy to mention here that it is a diffusive regime-driven process. Once Au-PEG clusters reach the cell membrane, they get spontaneously reduced close to membrane landmarks and makes bigger clusters via spontaneous aggregation which limits their further diffusivity, hence some of the reduced Au remain distributed around cell periphery. Evidence supporting this explanation that some portion of the gold may remain within the plasma membrane, is found in the enhanced Raman spectra. Both in whole cells (Fig. 3a, b) and the extracted cell fractions (Fig. 3c) of those cells treated with ionic Au-PEG clusters showed enhanced Raman signal in the nucleus and membrane. This Raman enhancement indicated that nanoparticles resided within the plasma membrane and the nucleus after the 4 h of treatment period. The concept that gold nanoparticles remain within the plasma membrane as the ionic Au-PEG clusters continue to migrate toward the nucleus is supported by the reduction of overall electron density observed as these clusters are fully internalized. After internalization through the plasma membrane the Au-PEG clusters undergo progressive reduction, collecting proteins from the cytoskeleton (SRCN1, ITGB1BP1, CALM1), cytosol (PRDX4, GBE1), endoplasmic reticulum (PRDX4, JKAMP), and lysosome (GM2A) regions. The proteins in these non-nuclear regions are involved in metabolic processes (GM2A, CALM1), response to protein unfolding and oxidative stress (JKAMP, PRDX4), cation binding (CALM1, ITG1BP1), intracellular signal transduction (ITGB1BP1), and Titin binding (CALM1)^59^. The protein ITG1BP1, is involved in intercellular signal transduction and cation binding, and is located within the plasma membrane, cytoskeleton, and nucleus^58^. The protein CALM1, involved in cation binding and titin binding (a nuclear protein)^58^. Both ITG1BP1 and CALM1 pose as likely potential moderators for migration of ionic Au-PEG clusters to the nucleus for terminal reduction because of their involvement in cation binding complementary with their subcellular locations and nuclear protein interactions. If ITGB1BP1 and CALM1 retain their normal protein function wherein they transduce signals to the nucleus through binding (while they are bound to the Au-PEG protein complex), they pose as a potential route for mediating the migration of the ionic Au-PEG clusters to the nucleus for terminal reduction. We ascertain that the ionic Au-PEG clusters migrate to the nucleus for terminal reduction because of the enhancement of the Raman (whole cell mapping and fractions) combined with the identification of proteins bound to gold that are exclusively found in the nucleus (DDX31, FRG1, TTN). These proteins, found exclusively within the nucleus, are involved in cation/calmodulin binding (TTN), actin filament binding (TTN, FRG1), ATP binding (TTN, DDX31), and nucleotide binding (DDX31, FRG1, TTN)^59^. The protein titin^60^, with at least 23 cation-binding sites as well as binding calmodulin, poses as a potential gateway for the ionic Au-PEG clusters’ entry into the nucleus and then to the proximity of other nucleotide binding proteins (DDX31, FRG1). This complete intracellular progressive reduction process, involving at least 16 different proteins localized throughout the cell without apparent protein degradation (Fig. 3d and Supplementary Fig. 3a–c), involving 287 different molecular processes (including nucleotide binding) with terminal reduction within the nucleus, poses as an interesting and innovative theranostic agent. Overall, the sequential bio-reduction process can be summerized as— (i) The first phase starts at extracellular regions where the proteins, IGLL5, IGHA1, ZG16B, AMY2B, GBE1, AMY2B, GBE1, and SCGB1D2, take part, where IGHA1 has been observed to be one of the prime protein responsible for the receptor-mediated phagocytosis of the Au-PEG clusters; (ii) The second phase starts within the plasma membrane where the proteins, IGHA1, CDH23, SRCN1, and ITGB1BP1 have taken part; (iii) Au-PEG clusters then undergo progressive reduction, collecting proteins from the cytoskeleton (SRCN1, ITGB1BP1, CALM1), cytosol (PRDX4, GBE1), endoplasmic reticulum (PRDX4, JKAMP), and lysosome (GM2A) regions and (iv) The migration of ionic Au-PEG clusters to the nucleus for terminal reduction occurs through the involvement of ITG1BP1 and CALM1. We have also ascertained DDX31, FRG1, TTN as the gold bound proteins those are exclusively found in the nucleus. This application, wherein nanoparticles are generated intracellularly through ionic Au-PEG clusters, would provide no plasmonic signal unless the treatment was successfully applied. From this Au-PEG application, gold nanoparticles generated intercellularly are bound to intact proteins involved in almost 300 different molecular processes (including nucleotide binding), and resulting nanoparticles localized within the nucleus. It may be presumed that protein corona would form as soon as the NPs were introduced into the biological milieu. Photothermal therapy can therefore potentially be applied using this process which would disrupt a host of molecular processes simultaneously as well as directly cause heating of the nucleus, nuclear proteins, and essentially nucleotides.

**Table 1:** Proteins adhered to intracellularly formed gold nanoparticles identified through LC–MS of the nuclear fraction of MCF-7 cells treated with ionic Au-PEG clusters.

**Fig. 5:**
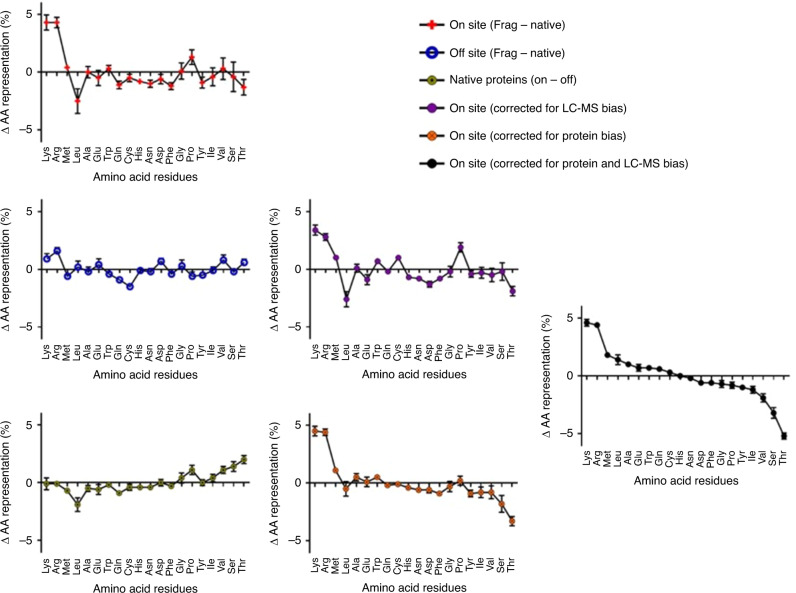
Changes in amino acid distributions from proteins identified through LC-MS. Amino acid distributions for on-site proteins between those found in LC–MS fragments and in nature (red); for off-site proteins between those found in LC–MS fragments and in nature (blue) representing the bias of the trypsin fragmentation and the LC–MS–MASCOT system; for native proteins between those found on- and off-site (yellow) representing the preference of the amino acid distribution between on- and off-site proteins; and various correction subtractions (purple or orange are partial corrections and black includes all corrections). Error bars are standard deviations of the mean (*n* ≥ 16 proteins).

To identify specific amino acids preferred in ionic gold reduction from these 16 proteins, we calculated the amino acid distribution in terms of overall percent for on-site protein fragments and off-site protein fragments and compared against the amino acid distribution of the native protein BLAST data from Uniprot. Further to identify which amino acids, in the identified proteins of interest, were preferentially interacting with and reducing gold, we made an assumption that amino acids which participate actively/directly in reduction would be bonded to the gold nanoparticles and in turn would be underrepresented in the LC–MS fragmentation analysis compared with the native AA distribution (Fig. 5). Comparing the change in distribution in AA representation in on-site proteins between fragment from LC–MS and whole native proteins (from Uniprot), we were able to determine which AAs were highly represented in the identified protein fragments (unlikely for participation in gold reduction [Lys]; [Arg]; [Pro]), and which were represented in fewer instances than in the native protein (likely candidate AAs are [Leu]; [Thr]). These bound amino acids would not appear in as high frequency as the uninvolved AAs through LC–MS analysis due to the random nature of the fragmentation combined with the gravimetric separation of protein fragments from dense gold nanoparticles. However, this AA distribution change (Fig. 5) does not account for the natural bias of the fragmentation process for LC–MS analysis. To account for the bias of the fragmentation process for LC–MS analysis, we would subtract the change in AA representation identified between fragments and whole native proteins identified off-site (bulk of the gel). Amino acids, which have a positive change in AA representation between fragments and whole native proteins identified off-site are considered overrepresented ([Arg]) by the fragmentation process and those with a negative change considered to be underrepresented ([Cys]). To correct for the bias of the fragmentation process, we would subtract this bias/favorability in AA representation. Now comparing the distribution change in AA representation in on-site proteins after subtracting bias from the fragmentation process, we were able to determine more reasonably [Lys], [Arg], and [Pro] as unlikely candidates for reduction of gold and [Leu] and [Thr] as likely candidates (Fig. 5). However, this AA distribution change with subtraction bias of the fragmentation process does not account for the variability of AAs between on-site and off-site proteins (Fig. 5). From comparing the AA distribution between on-site and off-site proteins in their native states, we identified AAs which more highly represented in the target than off-site proteins ([Pro]; [Val]; [Ser]; [Thr]) and AAs which had lower representation ([Leu]). After subtracting this bias of the AA distribution between native states of the on-site and off-site proteins, we were able to identify [Lys], and [Arg] as overrepresented (likely not involved in reduction), and [Ser] and [Thr] as underrepresented (likely candidates for gold reduction).

### In vitro impact

**Fig. 6:**
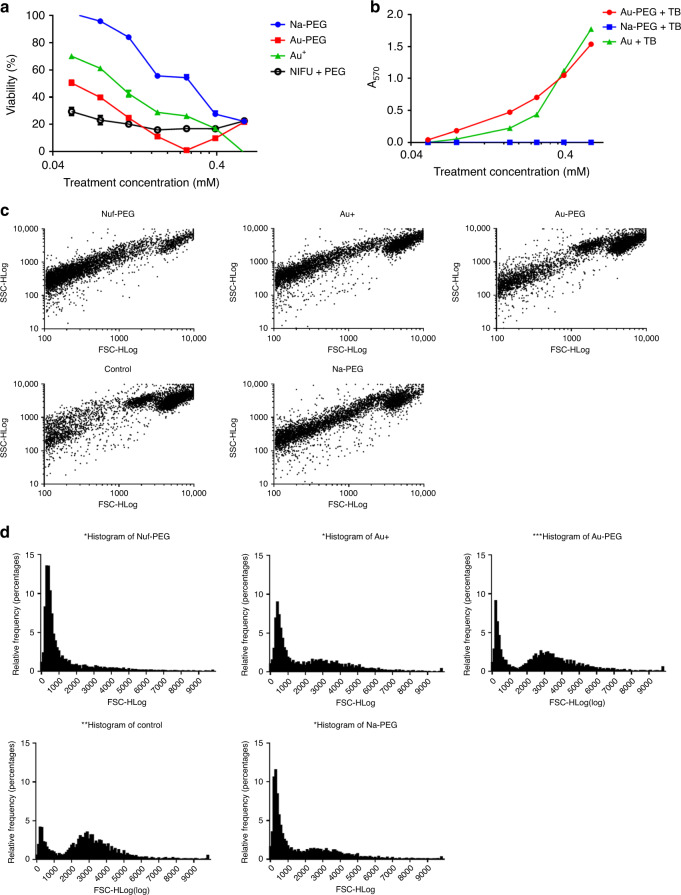
Cell viability of MCF-7 when treated with ionic gold. **a** Cells as assessed via thiazolium blue (MTT) metabolic assay for cells treated with Na–PEG (blue), Au^3+^ (green), Au–PEG (red) and nifuroxazide mixed with PEG (black). For (**a**) and (**b**), error bars are standard deviation of the mean (*n* = 3 biologically independent samples). Titrated light interactions between ionic-PEG mixtures (same color scheme) as admixed with thiazolium blue at concentrations used for MTT assay. Comparison of forward scatter vs side scatter plot for different samples at 0.24 mM treatments (**c**). Representative histogram analysis of different samples (**d**) showing two populations, one corresponding to dead cells at low FSC, whereas the second at higher FSC corresponding to healthy cells. Error bars in (**a**) and (**b**) are standard deviations of the mean.

To characterize the cytotoxicity of our ionic gold delivery platform, we compared apparent metabolic impacts (through MTT) and physical morphological changes (through flow cytometry without staining) against the treatments with ionic gold (without PEG), PEG with ionic sodium, and Nifuroxazide with PEG (NIFU-PEG). From the MTT assay, our intracellular gold nanoparticle generating solution (Au-PEG) appears to reduce cell viability at lower treatment concentrations compared with Au^3+^ and Na-PEG with a treatment-response dependent more on concentration than NIFU-PEG (Fig. 6a). However, there is an apparent inflection point in the apparent viability of Au-PEG-treated cells which causes us to question reliability of this assay for our treatment. This inflection point occurs in MCF-7 cells as the apparent cell viability approaches 0%. We do not believe that this inflection point in apparent cell viability reflects an actual resurgence in cell viability or a change in treatment behavior at higher concentration, but actually reflects a reaction between unreacted treatments (ionic gold) and the dye, Thiazolyl Blue Tetrazolium bromide. To support this hypothesis, that the unreacted ionic gold can react with the Thiazolium Blue, we incubated our cell treatments (Au^3+^, Au-PEG, and Na-PEG) at titrated concentrations with Thiazolium Blue (at MTT concentrations) and measure absorbance at the same wavelength used for the MTT assay (570 nm). From incubating our cell treatments with Thiazolium Blue in the absence of cells, we found a linear relationship between the concentration of ionic gold and the absorbance used to measure cell viability through MTT (Fig. 6b). From this we can ascertain that the inflection point in the cell viability is due to unreacted ionic gold interacting with the Thiazolium Blue. In addition to this, if our treatment can potentially interact with dyes, dye-based assays may not be ideal for characterizing the impact. On the other hand, by running flow cytometry and collecting forward scatter vs. side scatter measurements, we can comment on the morphological changes undergone by the cells in presence of various treatments without the use of dyes. From forward scatter parameter, one can monitor the change in cellular size, whereas from the side scatter parameter, one can comment on the internal complexity of cells. As shown in Fig. 6c, we observe three distinct populations/regions, marked as I (dead cells), II and III (cellular components) for all the samples including control. However, treatment with NIFU-PEG showed a marked increase of population of region 1 (dead cells) when compared with control, which could be directly attributed to the initiation of apoptosis by nifuroxazide resulting in cell death. Further histogram analysis via relative frequency vs. forward scatter (Fig. 6d) supplements this finding, we obtained two populations, the first one corresponding to dead cells having low diameter, whereas the second population bigger in diameter and corresponding to healthy cells. For control and Au-PEG treatments, we observed a smaller number of dead cells and a greater number of healthy cells. However, for NIFU-PEG, most of the population had smaller diameter corresponding to dead cells, and minimal population at the higher diameter (healthy cells) owing to the toxic nature of nifuroxazide.

A microarray analysis was used to identify changes in gene expression for pathways related to apoptosis (Supplementary Fig. 5) with directed focus on expression changes at the end of these pathways for pro-survival or pro-apoptosis. From this we found upregulation of PMAIP1, MCL1, DDIT3 (pro-apoptosis) together inhibiting the pro-survival gene BCL2 (reacts to calcium and iron) for Au-PEG treatments when compared with untreated MCF-7 cells. Additionally, the GADD45 (pro-survival) gene was highly upregulated (associated with DNA damage and growth arrest) for Au-PEG treatments.

### Investigation of internalization mechanisms

Cell internalization efficiency of our nanoparticles must be determined, so that we would be able to practically deliver high enough concentrations of gold for practical imaging purposes (X-Ray, photoacoustic, or fluorescence), and/or therapeutic photothermal ablation. To calculate the internalization efficiency, we treated MCF-7 cells in triplicate for 4 h with our ionic gold PEG clusters, collected the supernatant and cell pellet, submitted both for ICP analysis for gold and used the total mass of gold in the cell pellet vs. the supernatant to calculate the total internalized gold based on the treatment concentration. We found that nearly 50% of the gold was internalized in the breast cancer cells (Supplementary Fig. 6). We then investigated the internalization pathways those were involved using a series of inhibitors that would selectively block major endocytosis pathways (Supplementary Fig. 6). Small molecule inhibitors for our study selected in such a way that they did not exert any considerable toxicity by themselves in the short period of incubation. Furthermore, they were chosen so that they do not affect the actin cytoskeleton post treatment^23,61^. The working hypothesis of this study was based on the fact that a particular inhibitor would block a specific endocytic pathway and consequently not allow the nanoparticles to internalize in the cells via that particular pathway. A combination of 2-deoxyglucose (DOG) and sodium azide (NaN_3_) were employed to inhibit glycogenosis and cellular respiration, respectively, via energy-dependent uptake^62^. Chlorpromazine (CPM) is known to reduce the generation of clathrin-coated pits via a reversible translocation of clathrin and its adapter proteins from plasma membrane to intracellular vesicles. CPM was employed to understand the clathrin-dependant cellular internalization^63^. Dynamin (GTPase) dependency in endocytosis of cells was probed by using dynasore^64^. Additionally, nystatin, which is a sterol-binding agent, was used to study clathrin-independent inhibition of endocytosis. Nystatin is known cause dissembly of caveolae and cholesterol in the membrane^65,66^. We repeated our ICP analysis of internalized gold with these four inhibitors of various major pathways of cellular internalization. If the addition of any of these internalization inhibitors were to reduce the percent of treated gold found in the cellular pellet, we would interpret this as a pathway which our endocytosis was routed. From this study, however, we found that none of these inhibitory treatments were able to reduce the internalization of the gold (Supplementary Fig. 6). We found the lack of impact on internalization by these inhibitors perplexing, as these pathways were involved in majority of the cellular internalization and approximately half of the gold was being internalized. A microarray analysis was then used to identify changes in gene expression for pathways related endocytosis (Supplementary Fig. 7). Through this pathway map, we observed no change in the gene expression for clarthrin-dependent endocytosis (ARF6, PIPK1A, PLD1), clathrin-independent endocytosis (ARF6, SRC, PIP5K1A, HRAS, PLD1), or lipid raft formation (PIP2, MHC1, PIP3). This may indicate internalization through non-classical means or through mechanical forces generated as gold is reduced along the cell membrane forming bonds with cellular proteins. However, there are changes in gene expression for processes within this mapping. Within this map, genes those are the most heavily downregulated are associated with cation binding (TGFBR2, ERBB4, CBLB, GIT2), and those upregulated are associated with cell stress and protein refolding (HSPA1A). However, the participation of immunoglobulin heavy constant alpha 1 (IGHA1) protein during the bio-reduction of Au-PEG clusters to form AuNPs (Table 1) supports the receptor-mediated phagocytosis as one of plausible pathways of cellular internalization for these clusters.

### In vivo theranostics

**Fig. 7:**
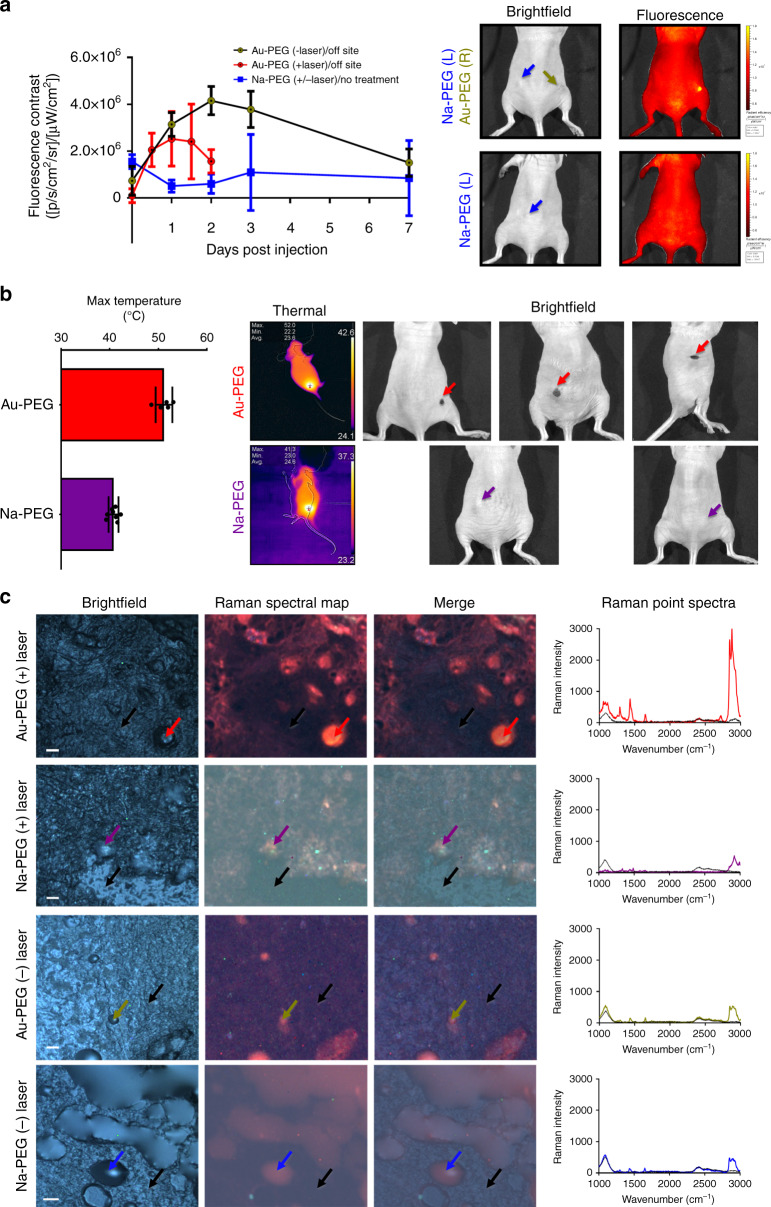
In vivo efficacy of the gold nanoparticles generated through vectorized biomineralization of ionic gold. **a** Fluorescence contrast afforded via in vivo imaging system (IVIS)-based imaging (430_Ex_ 840_Em_) between on-site and off-site across one week period for treatments of Au–PEG with no photothermal treatment (yellow), Au–PEG with photothermal treatment (red), or Na–PEG (blue) with bright field and fluorescence images representative of Au–PEG or Na–PEG-treated mice (three days post injection) with arrows indicating intratumoral injection sites for Na–PEG (blue) and Au–PEG (yellow). Error bars are standard deviation of the mean (*n* ≥ 3 biologically independent animals). **b** Photothermal effect of intratumorally generated gold nanoparticles via 3 min applications of 500 mW 633 nm laser as represented by maximum surface temperature from the tumors either treated with Na–PEG (purple) or Au–PEG (red) with thermal and bright field images representative of Na–PEG (purple) or Au–PEG (red)-treated mice after two laser applications (thresholding contrast for thermal images are automatically read via the iPad mounted FLIR software). Error bars are standard deviation of the mean (*n* ≥ 5 laser treatments). **c** Bright field, Raman spectral mapped and merged images of excised tumors from mice treated with Au–PEG (± laser ablation) or Na–PEG (± laser ablation) with corresponding Raman point spectra for positions designated with arrows. Scale bars are 20 µm.

From our application of ionic gold clustered with PEG, we have developed a unique system wherein gold nanoparticles are reduced by cellular biomolecules those are able to retain their functionality, including the capacity to guide the remaining cluster to the nucleus. The intracellular formation and nuclear migration of these gold nanoparticles presents a high potential for theranostic photothermal ablative application. To discover the theranostic potential of the intracellular generation of functional gold nanoparticles, we applied our ionic Au-PEG clusters to MCF-7 xenografts in J:NU mice compared with control injections of ionic Na-PEG (Supplementary Fig. 8). Intratumoral injections of ionic Au-PEG clusters presented fluorescence contrast via IVIS imaging with an excitation wavelength of 430 nm and an emission wavelength of 840 nm. Although some fluorescence signal at 430 nm excitation and 840 nm emission was observed, the lack of strong fluorescence signal in vitro was presumably due to the differences in microenvironment and cell density which could result in variation of sizes of nanoparticles as well as in concentration. This observed fluorescence emission is not uncommon for ultrasmall gold nanoclusters (<5 nm), wherein the emission wavelength can be tuned from 385 to 866 nm^67^. From our system, wherein gold nanoparticles were generated intratumorally, fluorescence contrast was observable above background as early as one day after injection of ionic Au-PEG clusters (Fig. 7a–c). We observed increasing contrast between injection sites and off-site locations for up to 2 days, which would then dissipate after one week (Fig. 7a). The increase in fluorescence contrast between the injection site and off-site locations over this 2-day period suggests that nanoparticles may be continually forming at the injection site for up to 2 days beyond the injection time point. This fluorescence contrast between injection site and off-site (no treatment) was not observed for ionic Na-PEG injections at all the time points. To investigate therapeutic potential, tumors injected with either Au-PEG or Na-PEG would receive laser treatments using 500 mW 633 nm continuous wave laser for 3 min (+laser) or 0 min (−laser) periods with an iPad-mounted IR camera to monitor and quantify the temperature changes. When applications of laser ablation were applied, tumor temperatures would rise to 51.2 ± 1.7 °C for successful intratumoral Au-PEG treatments and 40.8 ± 1.0 °C for Na-PEG treatments (Fig. 7b). Successful hyperthermia is commonly defined as heating tissue at >45 °C for several minutes (5–10 min)^68,69^. Lesions/scabbing and tumor remediation occurred for tumors with successful applications of Au-PEG with two or fewer laser applications in all but one mouse (*n* = 5). Laser applications made to successful Au-PEG treatments would reduce the apparent fluorescence contrast when compared against Au-PEG treatments without laser treatment (Fig. 7a). This apparent reduction in fluorescence contrast from laser treatment is likely due to the resultant hyperthermic process, either through loosening cell membranes and destroying tissue (allowing nanoparticle escape) and/or through the addition of thermal energy modifying the reaction kinetics (forming non-fluorescent nanoparticles). Hyperthermia causes cell damage by loosening cell membranes and denaturing proteins^70,71^, and hyperthermic laser applications have been demonstrated previously to spontaneously reduce nanoparticles through laser heating^72–74^. It is likely that a combination of these effects are taking place, wherein photothermal applications are simultaneously loosening tissue for the escape of fluorescent gold nanoparticles and unreacted ionic gold as well as initiating laser-induced gold nanoparticle formation (Supplementary Fig. 9). Applications of Au-PEG which were unsuccessfully applied intratumorally, due to the small size of the tumors (resulting in an intradermal application), would result in diffusive fluorescence with contrast between tumor and off-site no greater than Na-PEG (Supplementary Fig. 10). When ablative laser applications were applied to intradermal injections of Au-PEG (opposed to intratumoral injections), tumor temperatures would rise to 40.7 ± 2.3 °C (no significant difference than Na-PEG). The low fluorescence contrast and lack of photothermal effect from intradermal injections of Au-PEG (compared to intratumoral injections) provided us with near immediate feedback and utility of intratumorally generated gold nanoparticles towards theranostic application. This is because, after failed intratumoral injections, we were able to acquire fluorescent images for confirmation of the success of our application. This allowed us to predict if photothermal laser application would provide therapeutic effect or if we should rather wait for fluorescence to reduce background (by waiting one week) before attempting a second injection attempt. Therefore, if we applied laser therapy to unsuccessful injections, we would observe no hyperthermia impact (on-site or off-site). To further investigate the ionic Au-PEG cluster for theranostic applications focusing on the intratumoral generation of gold nanoparticles, organs (tumors, sentinel lymph nodes, liver, kidneys, and spleen) were harvested and imaged with either Raman mapping (Fig. 7c, Supplementary Fig. 11) or stained by H&E for bright field imaging (Supplementary Fig. 12). From Raman mapping, we found increased Raman signal indicating plasmonic enhancement in tumors treated with Au-PEG which had received photothermal ablation compared with the other treatments (Fig. 7c). This plasmonic enhancement, found only in the section of the tumor treated with Au-PEG clusters and laser therapy, indicates that photothermal ablation may induce or modify nanoparticle formation in a manner separate from the cellular reduction. The application of ionic Au-PEG clusters to generate fluorescent and photothermally active nanoparticles intratumorally resulted in no observable pathological effects through our short-term experiment regardless of the success of the administration. Additionally, we demonstrated that hyperthermic laser applications could be applied to modify the nanoparticle formation intratumorally.

## Discussion

In summary, we have demonstrated a simplistic nano-scaled delivery system that combines ionic gold and PEG, which enables the progressive reduction and formation of plasmonic gold nanoparticles. These nanoparticles are both fluorescent and provide a route for ablative therapies through photothermal heating. We have characterized this process through the identification of associated proteins combined with their entailing molecular processes and subcellular locations. We have determined that our process does not destroy the integrity of its associated proteins and requires considerably lower concentrations of ionic gold and time as compared to the previous literature methods. For this nanotheranostic platform, we understand that a cellular-driven synthesis may be perceived as a major drawback as it poses as an element outside of control. However, this lack in control may pose as a potential benefit wherein theranostic nanoparticles are generated differentially, dependent on the cellular machinery and microenvironment. Further to this, we envisage to observe this effect in normal control cells in comparison to the cancer cells and develop a more optimized generation of biomineralized plasmonic nanoparticles which can efficiently be used for hyperthermia applications in vivo. We envision future applications similar to this presented work, wherein the treatment focuses on optimization of the nanostructures to propel the next major advancement in nanomedicine. These envisioned fluid-form treatments will utilize and rely on cellular machinery dependent on the local pathology of the tissue providing highly specific opportunities for sensing, therapy, or control over local physiology.

## Methods

### Materials

Chloroauric acid (SKU: 254169-5G), bi-hydroxyl terminated polyethylene glycol (Mn = 10,000 g/mol) (SKU: 8218811000), sodium chloride (SKU: 746398-500G), βME (SKU: M6250-100ML), dimethyl sulfoxide (SKU: 276855-100ML), fetal bovine serum (SKU: TMS-013-B), thiazolyl blue tetrazolium bromide (SKU: M5655-500MG), phosphate buffered saline (SKU: P3813-10PAK), Dulbecco’s modified Eagle medium (SKU: D7777-10X1L), trypsin-EDTA (SKU: T4049-500ML), aluminum potassium sulfate dodecahydrate (SKU: A6435-100G), and sodium iodate (SKU: 71702-25G) were purchased from Sigma-Aldrich unless otherwise stated. All components for SDS–PAGE and protein extraction kit were purchased from BioRad Laboratories. Histology grade ethanol was purchased from Thermo Scientific. Histology grade xylene was purchased from VWR. Eosin Y Hematoxylin hydrate, and Phloxine B were purchased from Acros Organics.

### Au-PEG cluster synthesis

In a 20 ml scintillation vial, 0.015 mmoles of HAuCl_4_ and 500 mg of PEG (Mn = 10,000 g/mol) were mixed with 2 ml of carbon-filtered deionized water (0.2 µm cellulosic membrane, pH 4.0). This mixture was incubated in a 37 °C water bath for 30 min before being diluted for further use.

### Na-PEG cluster synthesis

In a 20 ml scintillation vial, 0.015 mmoles of NaCl and 500 mg of PEG (Mn = 10,000 g/mol) are mixed with 2 ml of carbon-filtered deionized water (0.2 µm cellulosic membrane, pH 4.0). This mixture was incubated in a 37 °C water bath for 30 min before being diluted 100 fold with carbon-filtered deionized water (0.2 µm cellulosic membrane, pH = 7).

### TEM measurements

For TEM, Au-PEG clusters were diluted 100-fold with carbon-filtered deionized water (0.2 μm cellulosic membrane, pH = 7) and 2.5 μl of the diluted Au-PEG clusters were placed on a 300-mesh carbon film supported by a copper grid and allowed to stabilize for 2 min. A filter paper was then used to remove liquid for thin film formation and then allowed to air dry while covered. Images were obtained using a Jeol 2010 cryo-electron microscope operating at 200 kV, and using different degrees of defocus to obtain an adequate bright contrast. Images were recorded on a Gatan UltraScan 2k × 2k CCD. These CCD images were processed and analyzed with ImageJ (http://rsbweb.nih.gov/ij/) version 1.48.

### Scanning electron microscopy and electron dispersion spectroscopy

For SEM-EDS, Au-PEG clusters were diluted 100-fold with carbon-filtered deionized water (0.2 μm cellulosic membrane, pH = 7) and several 3 μl drops of the diluted Au-PEG clusters were placed on a strip of copper tape placed on an SEM sample grid and allowed to stabilize for 5 min. A filter paper was used to remove liquid for thin film formation and then allowed to air dry while covered. Images were obtained using a Hitachi S4700 scanning electron microscope with Oxford Instruments ISIS EDS X-ray Microanalysis System. SEM images were captured using a 10 kV accelerating voltage, a 10 μA emission current, and a 12 mm working distance, adjusting sample height for coarse focus and low degrees of defocus to obtain secondary electron images and EDS. Images were recorded using a Centaurus BSE detector.

### UV–Vis absorption studies

For collecting the UV–Vis spectra, 1 ml of the as-prepared Au-PEG clusters were used. For calibration, the blank consisted of carbon-filtered deionized water (0.2 µm cellulosic membrane, pH = 7). The absorption spectra were acquired in scanning mode for the range of 200–1000 nm using a GeneSys 10 S UV–Vis spectrophotometer (Thermo Scientific, Rockford, IL). Measurements were taken at every 0.1 nm interval.

### Mammallian cell culture

MCF-7 cells (ER (+) human breast cancer cells) sourced from ATCC (catalog no. HTB-22) were cultured in Dulbecco’s modified Eagle’s medium (DMEM; Sigma) supplemented with 10% fetal bovine serum (FBS) and 1x Penstrep in T25 culture flasks (Cellstar, Germany) and were incubated at 37 °C in a 99% humidified atmosphere containing 5% CO_2_. Cells were regularly passaged by trypsinization with 0.1% trypsin (EDTA 0.02%, dextrose 0.05%, and trypsin 0.1%) in DPBS (pH 7.4). Non-synchronized cells were used for all the experiments.

### Cell growth inhibition studies with MTT assay

MCF-7 cells were seeded at 10,000 cells/well in DMEM with 10% FBS and 1x Penstrep (200 μl/well) in a 96-well plate and incubated at 37 °C in a 99% humidified atmosphere containing 5% CO_2_ for 24 h. Cell treatments were as follows: control, Na-PEG, nifuroxazide-PEG, Au-PEG, and Au^3+^. Each treatment of the cells had concentrations 0.05–0.6 mM. After treatment, cells were incubated for 44 h. After 44 h, 20 μl MTT solution (5 mg/ml Thiazolyl blue tetrazolium bromide) was added to each well and the plate was incubated for an additional 4 h. Media was then aspirated from each well and 200 μl dimethyl sulfoxide (DMSO) was added. The plate absorbance was then read at 570 nm wavelength using Gen5 Microplate Reader and Imager Software. For investigating the reaction with ionic gold and Thiazolyl blue tetrazolium bromide, identical concentrations were used, without cells.

### Cell culture treatment with Au-PEG clusters for transmission electron microscopy

Samples of Au-PEG were diluted in full cell media to 0.24 mM Au^3+^ by mixing 0.5 ml Au-PEG clusters with 15 ml cell media. After removing old media, this mixture was incubated on a monolayer of MCF7 cells (grown in T-25 flasks until ~80% confluency) growing at 37 °C with 5% CO_2_ for 4 h. After this incubation period, growth medium was discarded and cell monolayer was washed with DPBS before trypsinizing the treated cells. Cells were harvested in small 1.5 ml centrifuge tubes and collected in DPBS before fixing. After the cell pellet was fixed in a Karnovsky’s fixative in phosphate buffered 2% glutaraldehyde and 2.5% paraformaldehyde. Microwave fixation was used with this primary fixative, and the tissue is then washed in Sorenson’s phosphate buffer with no further additives. Microwave fixation was also used with the secondary 2% osmium tetroxide fixative, followed by the addition of 3% potassium ferricyanide for 30 min. After washing with water, saturated uranyl acetate was added for enbloc staining. The tissue was dehydrated in a series of increasing concentrations of ethanol. Acetonitrile was used as the transition fluid between ethanol and the epoxy. Infiltration series was done with an epoxy mixture using the epon substitute Lx112. The resulting blocks were polymerized at 90 °C overnight, trimmed and ultrathin sectioned with diamond knives. Sections were stained with uranyl acetate (2%) and lead citrate (3%) and examined or photographed with a Hitachi H600 transmission electron microscope at 75 kV.

### Enhanced darkfield hyperspectral imaging

Optical and hyperspectral images were captured at ×60 magnification under enhanced darkfield illumination using the CytoViva hyperspectral imaging system (Auburn, AL). This hyperspectral imaging system couples an enhanced dark-field illuminator with optical and hyperspectral CCD cameras. The hyperspectral images, also called datacubes, were collected using the “push broom” method. The spectral data (400–1000 nm) was acquired 1 pixel row at a time. The acquisition was facilitated by a motorized stage. The hyperspectral analysis software ENVI compiled this spectral and spatial data into a datacube, in which each pixel contained spectral data.

Spectral libraries corresponding to the reduced gold nanoparticles were built from the images of exposed MCF-7 cells. These libraries were filtered against a negative control image (cells only, Supplementary Fig. S1e) to ensure no false-positive mapping of the nanoparticles. Using the spectral angle mapper (SAM) algorithm, the spectral libraries were compared to their respective images.

### Control treatments with Au-PEG clusters

Both 150 µl of as-prepared Au-PEG clusters and 250 µl of media either fresh or having been used to incubate cells for ~24 h (‘spent’ media) were mixed in a 2 ml microcentrifuge tube and incubated in a water bath for 24 h. From these samples, 2.5 µl was collected for TEM analysis as described in the “TEM measurements” section.

2 ml of as-prepared Au-PEG clusters were added to an empty poly-l-lysine-treated culture plate. This container was incubated at 37 °C in a 99% humidified atmosphere containing 5% CO_2_ for 24 h. From these samples, 2.5 µl would be extracted for TEM analysis as described in the TEM measurements section.

### Cell culture treatment with Au-PEG clusters for Raman spectroscopic analysis

Samples of Au-PEG were diluted in full cell media to 0.24 mM Au^3+^ by mixing 0.5 ml Au-PEG clusters with 15 ml cell media. After removing old media, this mixture would incubate on a monolayer of MCF7 cells (grown on glass slides until ~80% confluency) growing at 37 °C with 5% CO_2_ for 4 h. After this incubation period, growth medium was discarded and cell monolayer was washed with DPBS before fixing with 37% formaldehyde solution. Raman spectra were acquired on cells incubated with Au-PEG treatment and on control cells lacking treatment in reflection mode (LabRAM HR 3D Horiba confocal microscope). Laser light was focused through a ×100, NA 0.8 objective into the sample plane and the scattering was collected in the reflection geometry using the spectrograph coupled with an Andor Newton back-illuminated EMCCD camera. The excitation wavelength for the measurements was set to 633 nm, and the power was set to 8 mW at the sample with a 0.2 s acquisition time. Raman shift from 1000 to 3050 cm^–1^ was collected at 8 cm^–1^ spectral resolution. Intensities of select vibrational modes were selected to generate the Raman images.

### Cell fractionation of treated cells

Samples of Au-PEG were diluted in full cell media to 0.24 mM Au^3+^ by mixing 0.5 ml Au-PEG clusters with 15 ml cell media. After removing old media, this mixture was administered on a monolayer of MCF7 cells (grown in T-25 flask ~80% confluency) growing at 37 °C with 5% CO_2_ for 4 h. The cells were then trypsinized and centrifuged at 20,000 × *g* to form a cell pellet. The trypsin was removed, and the pellet suspended in 1.5 ml DPBS before tip sonication (5 s ON, 2 s OFF at 1 A for 2 min) was used to rupture the cells. Ruptured cells were separated into cell fractions^1^ and homogenate was first centrifuged at 600 × *g* to pellet unbroken cells. Supernatant was collected and centrifuged at 15,000 × *g* for 5 min. The pellet was separated and the supernatant was collected and centrifuged at 100,000 × *g* for 60 min. The pellet was separated and the supernatant was collected and centrifuged at 300,000 × *g* for 120 min.

Using an Eppendorf (Hamburg, Germany) 5424R microcentrifuge for centrifugation speeds lower than 100,000 × *g*, and a Beckman Coulter (Indianapolis, IN) Optima MAX-XP Ultracentrifuge for speeds above 100,000 × *g*. Pelleted fractions were suspended in 1 ml DPBS (pH 7.4) before SDS–PAGE or Raman analysis. SDS–PAGE and Raman samples were prepared immediately after fractionization.

### Raman spectroscopic analysis

Raman measurements were taken using a Nanophoton Raman instrument at Frederick Seitz Materials Research Laboratory Central research facilities, UIUC (532 nm laser). For each spectrum, a grating (600 mm^−1^) scan was taken over the range of 1000–3000 cm^−1^ at 0.2% laser power for 1 min (with ×20 objective). An average of 20 spectra was recorded per sample. Spectra were exported as.txt files and plotted using Graphpad prism.

### SDS–PAGE analysis of cell fractions

A BioRad Mini-PROTEAN^®^ tetra vertical electrophoresis cell was loaded with 2 × 5–20% gradient Mini-PROTEAN gels and the electrophoresis cell filled to the appropriate fill line with 1x Tris/Glycine SDS buffer. Two portions of nuclear protein fractions from cells treated with Au-PEG, Na-PEG, or untreated were admixed with 4x Laemmli sample buffer (BioRad) either containing 2.5% βME or with 1x Tris/glycine SDS buffer in the same volume with pipetting to addmix. From these prepared protein samples 20 µl was added to wells 2–4 and 6–8, with 5 µl of Precision Plus (10–250 kDa) protein ladder in lanes 5 and 9. After allowing the samples to settle in their respective wells for 5 min the cover of the electrophoresis cell was added, power turned on to 200 V and allowed to run ~35 min (the dye front <1 cm from the edge of the gel). For LC–MS, the nuclear fraction extracted from cells treated with Au-PEG were allowed to run for >3 h (until all of the ladder proteins had run off the gel) without protein from other cell treatments.

### Coomassie Blue total protein stain

Gel was briefly rinsed with DI H_2_O before being immersed in protein fixing solution (10% acetic acid, 10% methanol, in Di H_2_O) for 30 min with gentle rocking. After fixing for 30 min, the gel was stained with 1x Coomassie Blue staining solution (BioRad) for 2 h with gentle rocking. After staining, the gel was submerged in destaining solution (10% acetic acid in DI H_2_O) for 24 h, replacing the destaining solution as it became saturated with residual Coomassie stain. After the gel was adequately destained, a BioRad gel-dock imaging system was used to image the stained gel. The images were contrast enhanced, and band intensities analyzed with ImageJ. The uncropped images for these coomassie-stained SDS–PAGE gels have been shown in Supplementary Fig. 3.

### Protein extraction via passive elution and LC–MS analysis

In order to separate proteins, bound to gold nanoparticles from unbound, we used the nuclear fractions from MCF-7 cells treated with Au-PEG and allowed it to run through gel electrophoresis in excess of time (>3 h). The top portion of the gel containing the wells of the gel (the top 1.5 cm) were excised from the bulk of the gel with a scalpel and placed into separate 15 ml centrifuge tubes. The gel pieces were ground inside their respective containers with sterile cleaned metal weighing spatulas before mixing with minimal elution buffer (50 mM Tris–HCl, 150 mM NaCl, and 0.1 mM EDTA; at pH 7.5), but ensuring that the gel pieces were completely immersed. These immersed gel fragments containing protein were incubated at room temperature on a rocker for 48 h. After incubation, samples were centrifuged at 5000 × *g* for 10 min before removing the supernatant for LC–MS analysis via partial trypsinization digestion performed in the Roy J. Carver Biotechnology Center (CBC).

### Inhibitor study

A T-175 flask of MCF7 cells at 80% confluency was split and plated (at equal cell density) into 15 T-25 culture flasks. Cells were grown for 24 h before being incubated with various endocytic inhibitors. Inhibitor formulations were made with reconstituted medium containing NaN_3_ and deoxyglucose, CPM, nystatin and dynasore at a concentration of 10, 50, 28 μM, 180 nM and 80 μM, respectively^2–4^. Cells were incubated with inhibitors for 1 h at ambient condition. Inhibitors were then replaced with Au-PEG diluted in full cell media to 0.24 mM Au^3+^ by mixing 0.5 ml Au-PEG clusters with 15 ml cell media. All the treatments were performed in triplicate. Cells treated with ionic Au-PEG clusters in absence of inhibitors were used as control. After the required incubation time of 4 h, cell pellets and supernatants were collected for each sample and subjected to further ICP-MS analysis to determine the intracellular amount of gold.

### Flow cytometry analysis

To further characterize the cytotoxicity of our ionic gold delivery platform, we performed flow cytometry analysis. Cells (MCF-7, 10,000 cells/well) were grown in 24-well plate for 24 h before treating with different samples prepared for this study for 4 h. At the end of incubation, cells were trypsinized and collected in 0.2% FBS containing DPBS. Samples were analyzed using a Guava EasyCyte Plus Flow cytometer. For each sample, data from 5000 single cell events were collected for 3 min, in triplicates, and forward scatter vs. side scatter information were recorded. The results were analyzed and plotted using Flowpy.

### Animal studies

To investigate potential theranostic applications pre-clinically, we generated and treated MCF-7 xenograft tumors in female J/NU immunodeficient double knockout mice. The experimental protocol received ethical approval and was approved by the Institutional Animal Care and Use Committee (IACUC), University of Illinois at Urbana–Champaign, and satisfied all University and National Institutes of Health (NIH) rules for the humane use of laboratory animals. All the animal experiments were complied by the relevant ethical regulations for animal testing and research and carried out in accordance with the approved guidelines. Female J/Nu mice (2–3 weeks old) were bought from Jackson Laboratories, USA. Upon arrival, mice were allowed 2 weeks for acclimation. Animals were group housed with free access to food and water, and individuality marked through ear punching. We injected 10^6^ cells in matrigel on the hind flanks of *n* = 12 mice with 1 injection per side. Of these injections, 16 tumors grew to usable size (~5 × 5 mm^2^). There were 5 mice with 2 tumors, 6 with only 1, and 1 mouse which did not have any tumors of usable size. These 16 tumors received intratumoral injections of either Na-PEG or Au-PEG. Mice which had tumor growth to suitable size (~5 × 5 mm^2^) on both flanks received two different treatments to both tumors (i.e. Au-PEG or Na-PEG and ±laser ablation). Tumors injected with either Au-PEG or Na-PEG received photothermal ablative therapy using 500 mW 633 nm continuous wave laser for 5 min (+laser) or 0 min (−laser) periods with IR camera (FLIR technologies) to monitor and quantify the temperature changes. From these groups, we had four tumors with Na-PEG injections and 0 min of laser treatment; three tumors with Na-PEG injections and 5 min of laser treatment; four tumors with Au-PEG injections and 0 min of laser treatment; five tumors with Au-PEG injections and 5 min of laser treatment. For all tumors which were successfully ablated, treatments were no longer made for those mice. Injections of 40 µl were administered using 32 gauge syringe needles over 2 min under isoflurane anesthesia (2.5% in oxygen) with an oxygen flow rate of 1 l/h.

### IVIS imaging studies

Animals were serially imaged at 1, 24, 48, 72, and 168 h after treatment at 430 nm excitation and 840 nm emission wavelengths. Animals which received photothermal ablative intervention were imaged at these same time points and additionally 30 min after laser treatment (1.5, and 24.5, and 48.5 h). During imaging, animals were anesthetized with isoflurane (2.5% in oxygen) with an oxygen flow rate of 1 l/hr.

### Photothermal ablative therapy with IR-Imaging

Photothermal ablative therapy was applied using 500 mW 633 nm continuous wave laser (RLTMRL-635-500-5, Roithner Lasertechnik) with 40 mm^2^ beam diameter for 5 min (+laser) or 0 min (−laser) periods and monitored with IR camera (FLIR technologies) to visualize and quantify the temperature changes. Anesthesized mice were placed on the laser stand mount, using a machine-etched crosshair, so that the beam path would intersect directly with the tumor. Upon completion of the laser therapy, a neutral density filter with 100% OD was placed in front of the beam, blocking the path of the laser. See Supplementary Fig. S9 for laser stand setup. An iPad-mounted IR-camera (FLIR Technologies) was used to obtain snapshot thermal images at 2.5 min after photothermal applications with a laser. Temperature maxima for these images were considered as photothermal treatments representative of surface temperature. During photothermal treatments and IR-imaging animals were anesthetized with isoflurane (2.5% in oxygen) with an oxygen flow rate of 1 l/h.

### Histology sectioning

At the end of the experiment, animals were sacrificed via anesthesia overdose. Organ tissues (tumors, sentinel lymph nodes, liver, kidneys, and spleen) were immediately excised and frozen in cassettes containing optimal cutting temperature (OCT) compound. Embedded tissue blocks were clamped in microtome and sectioned at 6 μm thickness. Tumor and sentinel lymph node slices were either left unstained (for Raman mapping) or stained with hematoxylin and eosin (H&E) for bright field imaging.

### H&E staining

Slides were first fixed for 5 min in 4% PFA. Immediately following fixation, slides were rinsed in cold PBS. Slides were then stained with H&E using standard protocol. Briefly, slides were immersed for 8 min in Mayer hematoxylin solution, followed by a 2 min wash in warm, slow running water. Then, slides were blued with two dips in lithium carbonate. Slides were again washed in warm slow running water. Slides were then immersed in 80% ethanol for 2 min, followed by 5 min in eosin. Slides were dehydrated by performing sequential immersions in ethanol, 4 ×2 min in 95% ethanol (in two separate containers), 4 × 2 min in 100% ethanol (in two separate containers), and finally 3 × 3 min immersions in xylene (in three separate containers). Coverslips were then applied using xylene-based Permount.

### Raman-mapped histology

Unstained tissue sections were imaged in the reflection mode via LabRAM HR 3D Horiba confocal microscope with 532 nm wavelength excitation laser with power set at 8 mW. The laser light was focused through a LWD Visible ×20, NA 0.40, objective into the sample plane and scattering was recorded in the reflection geometry using a spectrograph coupled with an Andor Newton back-illuminated EMCCD camera. An acquisition time of 1 s was used. The Raman shift from 310 to 3410 cm^–1^ was recorded with a spectral resolution of 3 cm^–1^.

### Reporting summary

Further information on research design is available in the Nature Research Reporting Summary linked to this article.

## Supplementary information

Supplementary Information

Reporting Summary

## Data Availability

The authors declare that the data supporting all the findings of this study are available within the article and Supporting Information files. Zip files for Microarray data can be downloaded using the following link (https://uofi.box.com/s/b10c77c0anr5q4v7sm9k313w5r8fc92z); CSV files for protein MS data can be downloaded using the following links: Top gel portion (https://uofi.box.com/s/9l6j732etrh2uqy17o0f3p8aqvp5p15a); Bottom gel portion (https://uofi.box.com/s/xjb209go4okmvba69bx405wegrv3rdmf). All the other relevant data are available from the author upon reasonable request.
