# Supplementary material for: Intratumoral generation of photothermal gold nanoparticles through a vectorized biomineralization of ionic gold
Source: Nat Commun. 2020 Sep 10;11:4530. doi: 10.1038/s41467-020-17595-6 (PMC7483505; doi:10.1038/s41467-020-17595-6)
Supplement: Supplementary file 1 — Supplementary Information [file 41467_2020_17595_MOESM1_ESM.pdf]

Supplementary Information

Intratumoral Generation of Photothermal Gold Nanoparticles through a Vectorized Biomineralization of Ionic Gold

Aaron S. Schwartz-Duval[1,2,3], Christian J. Konopka[1,2], Parikshit Moitra[4,5,6], Enrique A. Daza[1,2,3], Indrajit Srivastava[1,2,3], Elyse V. Johnson[7], Taylor L. Kampert[1,2,3], Stanley Fayn[1,2], Anand Haran[1,2], Lawrence W. Dobrucki[1,2], and Dipanjan Pan[1,2,3,4,5,6,*]

[1] Department of Bioengineering, University of Illinois, Urbana-Champaign, Urbana, 61801, USA

[2] Beckman Institute, University of Illinois, Urbana-Champaign, Urbana, 61801, USA

[3] Biomedical Research Center, Carle Foundation Hospital, Urbana, IL, USA

[4] Department of Diagnostic Radiology and Nuclear Medicine, University of Maryland Baltimore School of Medicine, Baltimore, Maryland, 21201, United States

[5] Department of Pediatrics, Center for Blood Oxygen Transport and Hemostasis, University of Maryland Baltimore School of Medicine, Health Sciences Research Facility III, 670 W Baltimore St., Baltimore, Maryland 21201, United States

[6] Department of Chemical, Biochemical and Environmental Engineering, University of Maryland Baltimore County, Interdisciplinary Health Sciences Facility, 1000 Hilltop Circle Baltimore, Baltimore, Maryland 21250, United States

[7] Cytoviva inc., Auburn, 36830, USA

*To whom correspondence should be addressed: Prof. Dipanjan Pan; E-mail: dipanjan@som.umaryland.edu

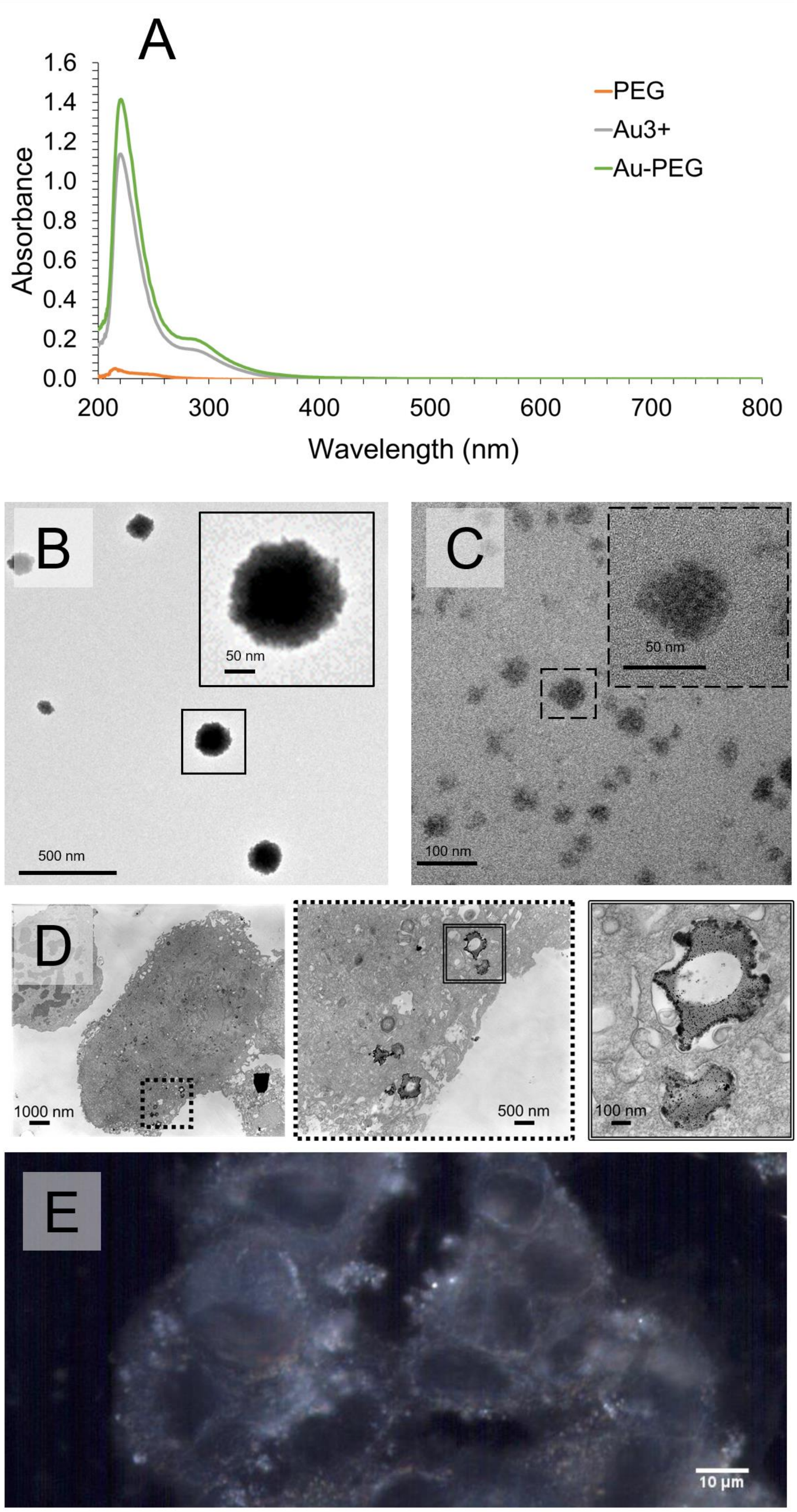

Supplementary Figure 1. Characterization of ionic Au-PEG cluster: (A) UV-Vis absorbance spectra of ionic Au-PEG clusters (200-800 nm) as well as it's individual components at identical

concentrations. TEM images from the extracted solution of ionic Au-PEG treatments to cell culture plates with a magnification of Au-PEG cluster (B), and fresh cell culture media incubated with Au-PEG clusters (C). Additional TEM images of Au-PEG treated MCF-7 cells at various magnifications (D). Enhanced hyperspectral darkfield image of untreated MCF-7 cells used for negative filtering for Figure 2A and 2B (E).

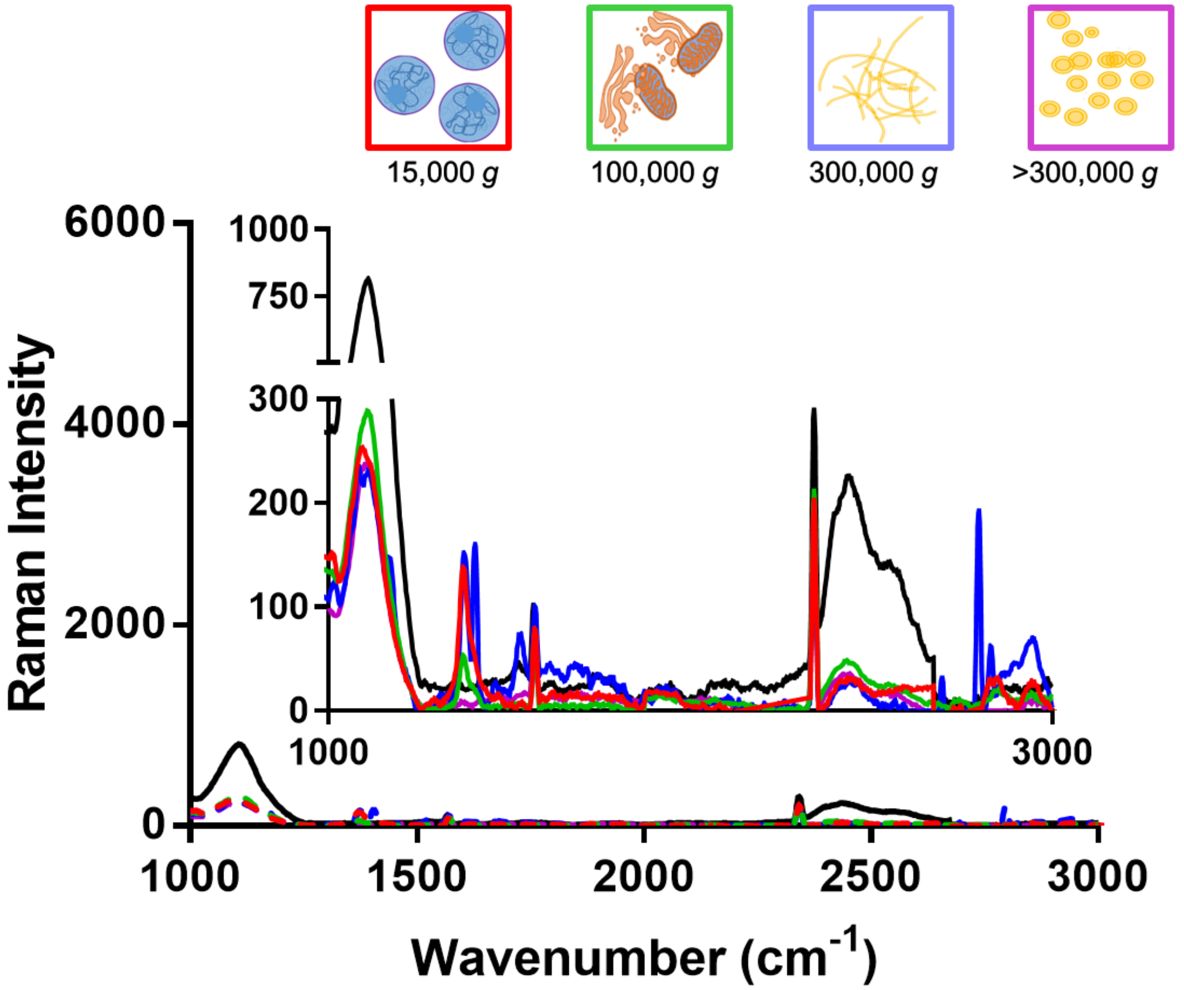

Supplementary Figure 2. Raman spectra of cellular fractions (all from MCF-7 cells): Nucleus and large organelles, 15,000 *g* (Red); mitochondria, lysosomes, and other medium sized organelles, 100,000 *g* (Green); membrane fragments, 300,000 *g* (Blue); and highly soluble/cytosolic molecules (Violet), >300,000 *g*; and the glass slide without cell fractions (Black).

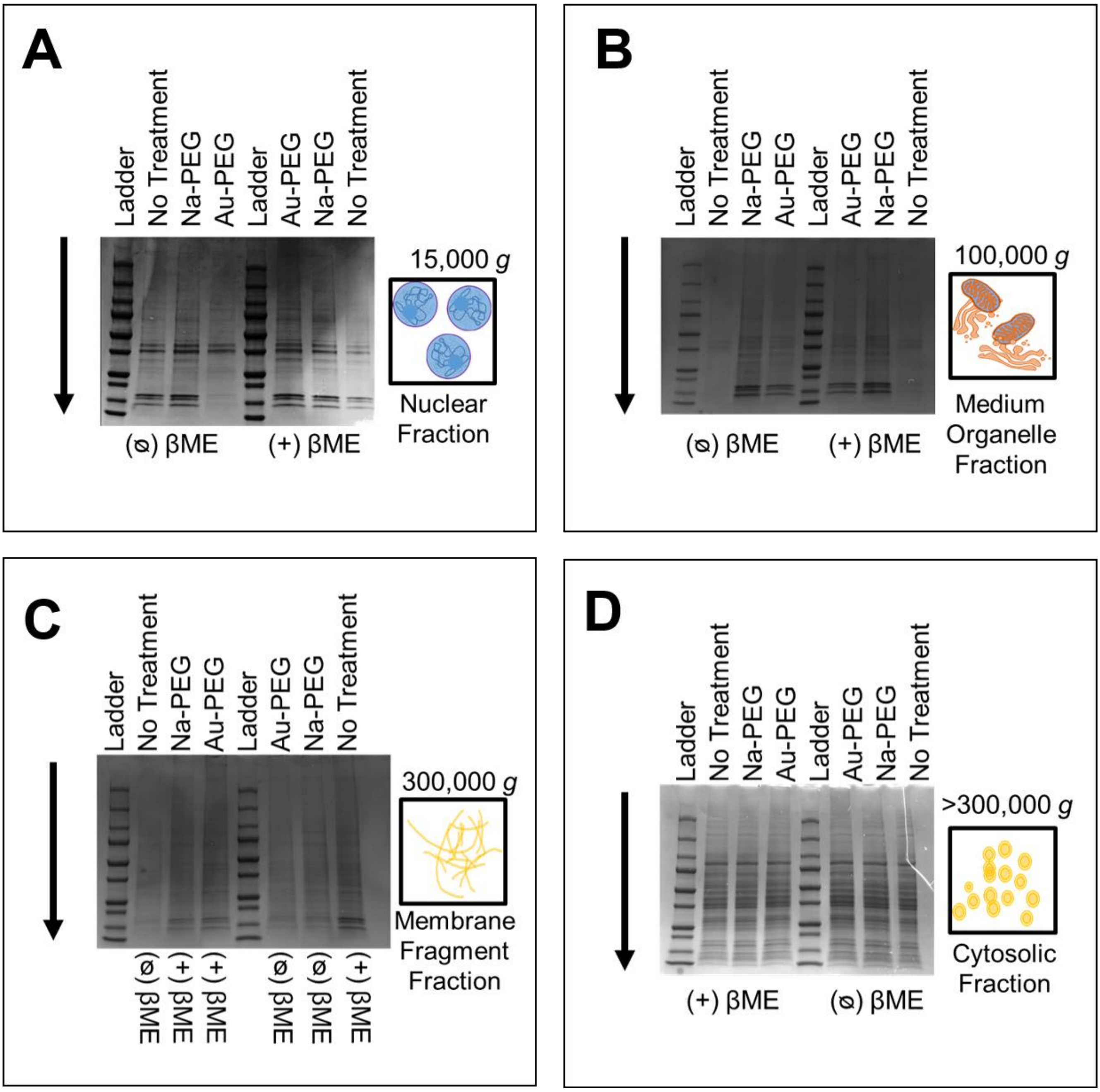

Supplementary Figure 3. Confirmation of intracellular reduction by SDS-PAGE: SDS-PAGE stained with Coomassie Blue of cellular fractions including (A) nuclear, 15,000 *g*; (B) medium organelle, 100,000 *g*; (C) membrane fragments, 300,000 *g*; and (D) cytosolic, >300,000 *g* regions of MCF-7 cells untreated, treated with Na-PEG, or Au-PEG with either standard SDS-PAGE conditions [(+) βME], or without β-mercaptoethanol [(⊘) βME]. Arrows indicate direction of protein migration due to electrophoresis.

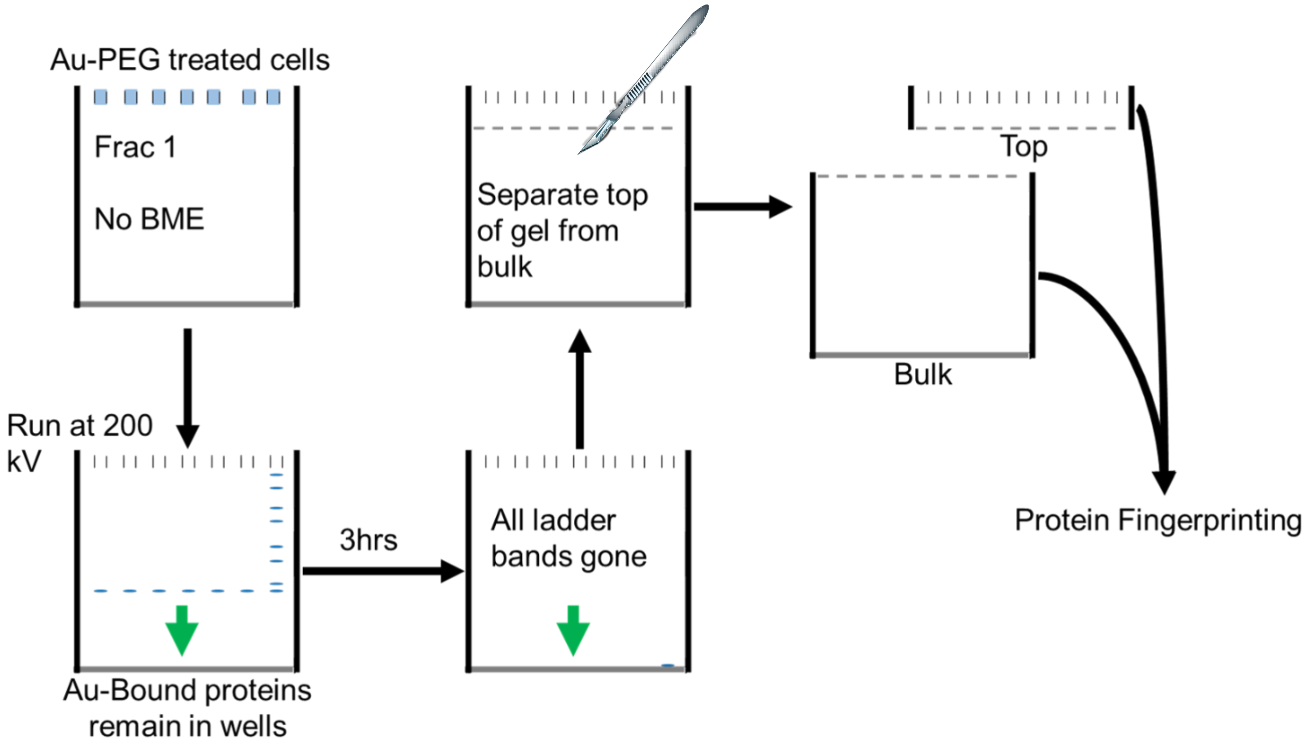

Supplementary Figure 4. Schematic for separation of Au bound proteins from the non-bounded ones: The process describing the separation of Au-bound proteins from non-bound ones through gel electrophoresis, followed by separation of portions of the gel for LC-MS analysis.

Protein Name	Subcellular Location						
	extracellular or secreted	plasma membrane	cytoskeleton	endoplasmic reticulum	lysosome	cytosol/ cytoplasm	nucleus
Zymogen granule protein 16 homolog B	■						
Secretoglobin family 1D member 2	■						
Immunoglobulin lambda-like polypeptide 5	■						
Alpha-amylase 2B	■						
Immunoglobulin heavy constant alpha 1	■	■					
1,4-alpha-glucan-branching enzyme	■					■	
Cadherin-23		■					
SRC kinase signaling inhibitor 1		■	■				
Integrin beta-1-binding protein 1		■	■				■
Peroxiredoxin-4				■		■	
JNK1/MAPK8-associated membrane protein				■			
Ganglioside GM2 activator					■		
Calmodulin-1			■				
Titin							■
Protein FRG1							■
Probable ATP-dependent RNA helicase							■

Supplementary Table 1. Proteins identified from the LC-MS analysis and the subcellular regions in which they are located, as annotated on UniProt.org through COMPARTMENTS.[1]

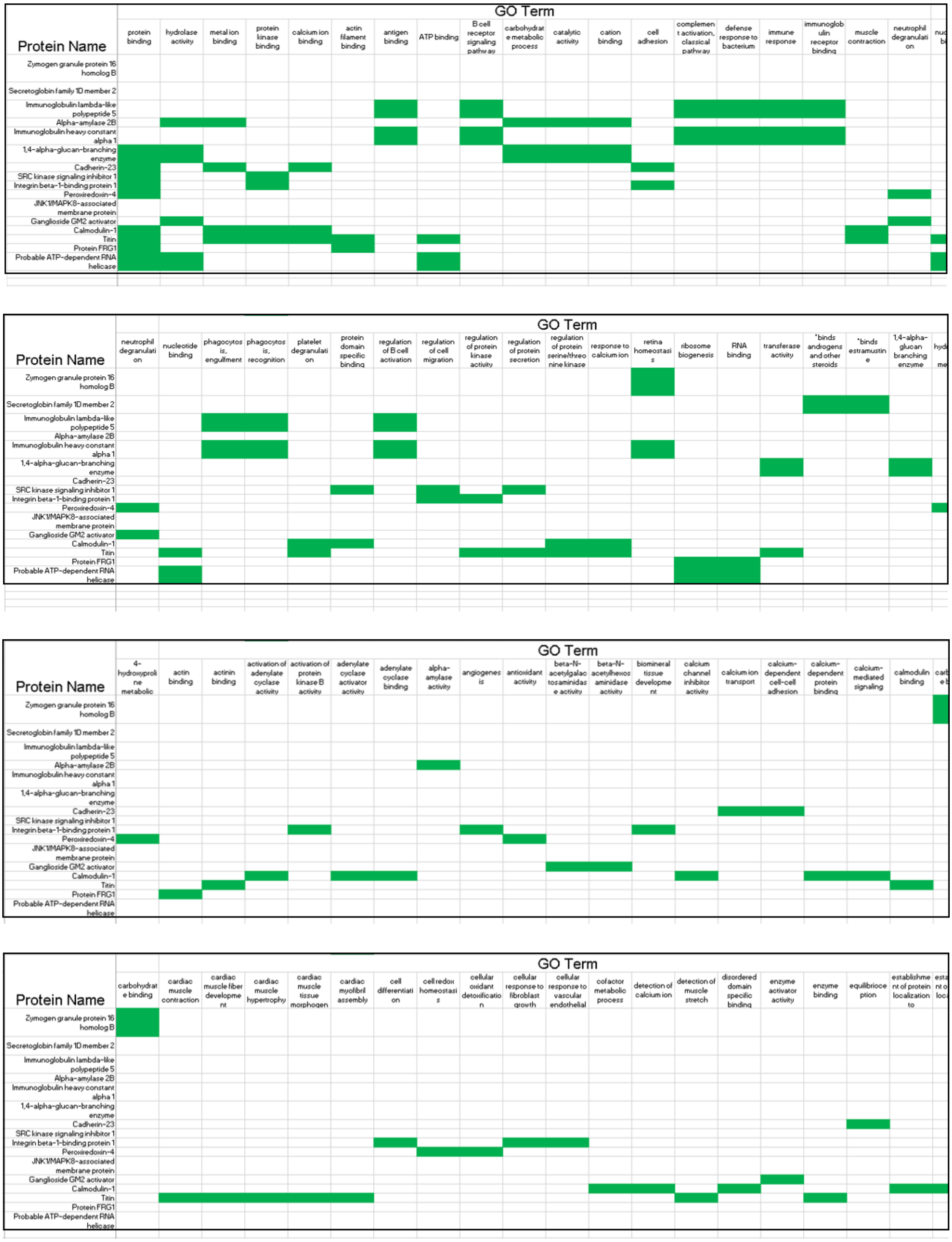

Supplementary Table 2. Proteins identified from the LC-MS analysis and the molecular processes in which they are involved, as annotated on UniProt.org through phylogenetic-based propagation of functional gene ontology (GO) annotations.[2]

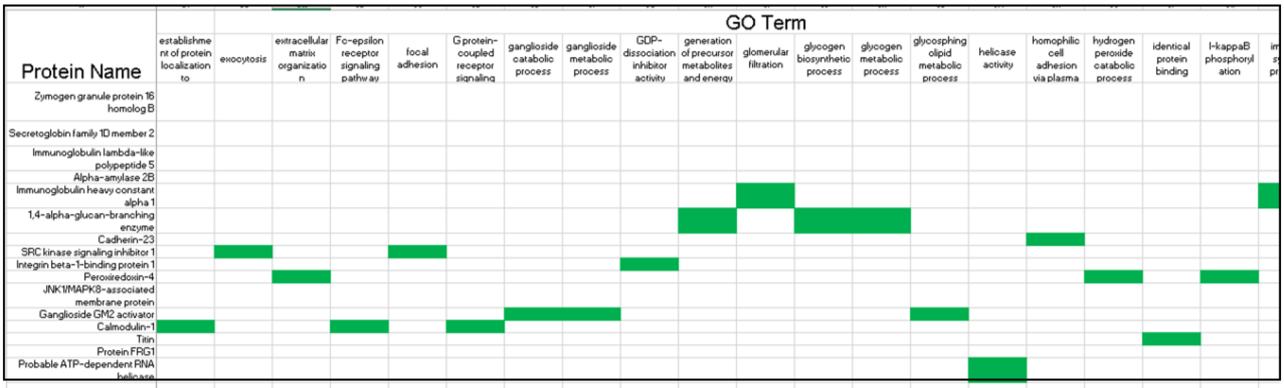

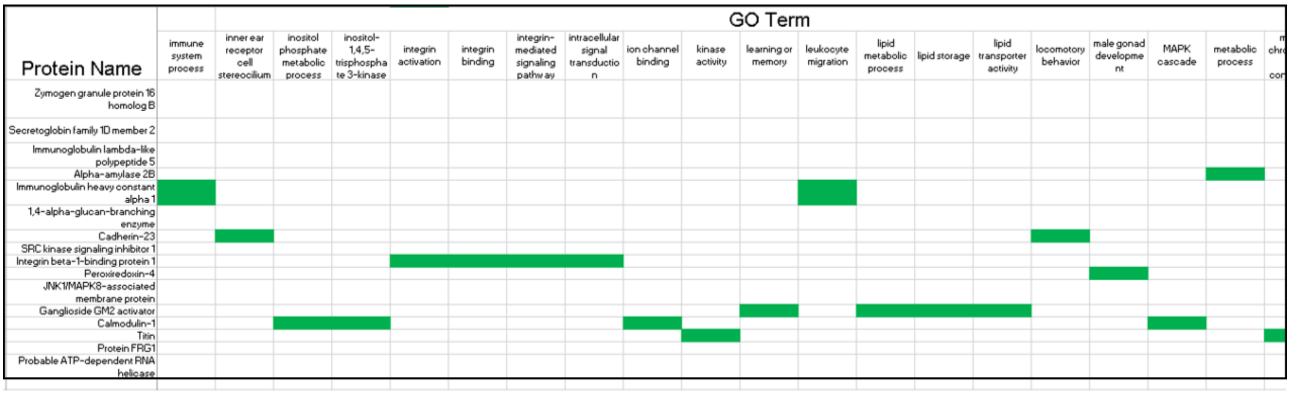

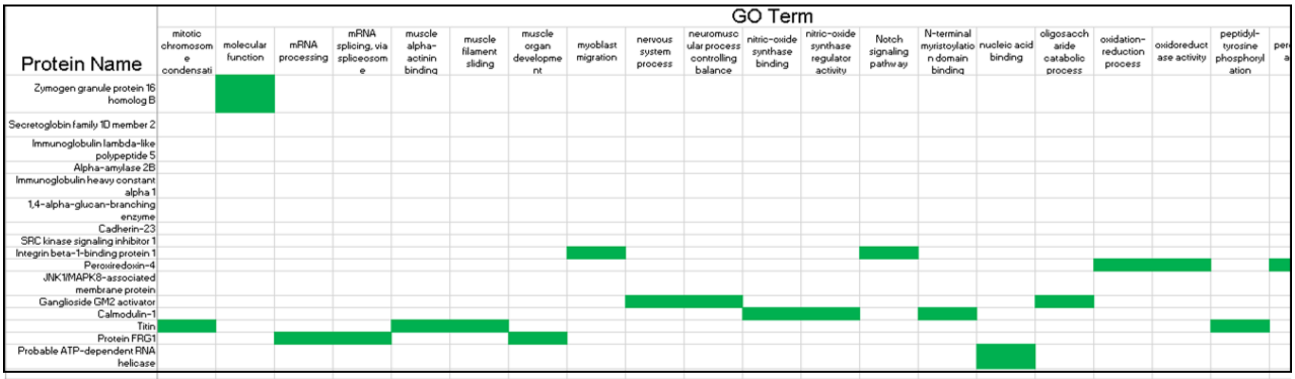

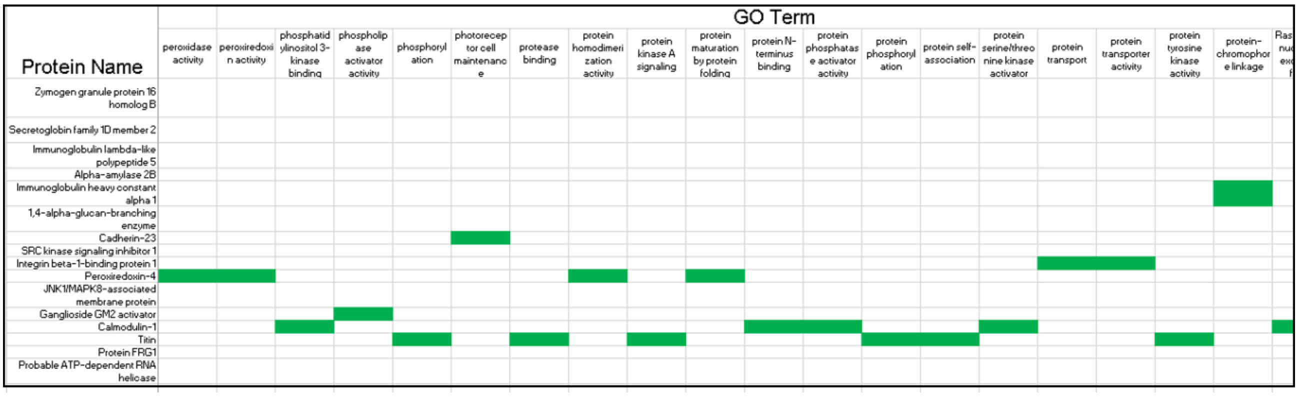

Supplementary Table 2 contd. Proteins identified from the LC-MS analysis and the molecular processes in which they are involved, as annotated on UniProt.org through phylogenetic-based propagation of functional gene ontology (GO) annotations.[2]

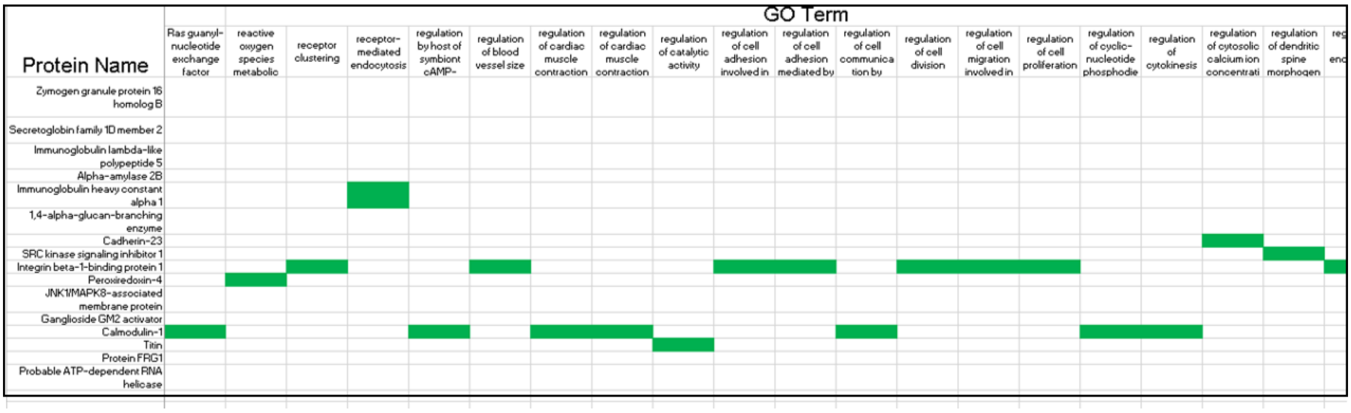

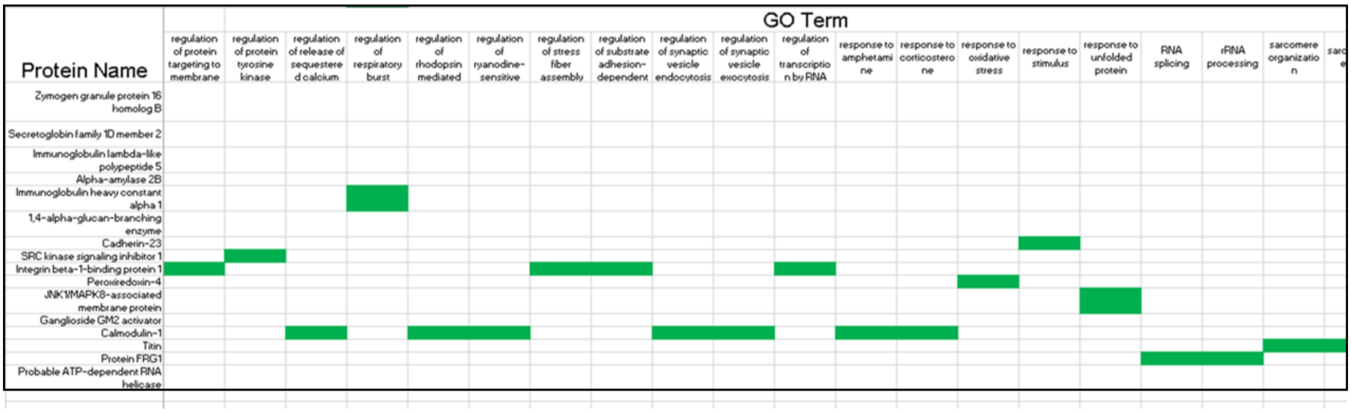

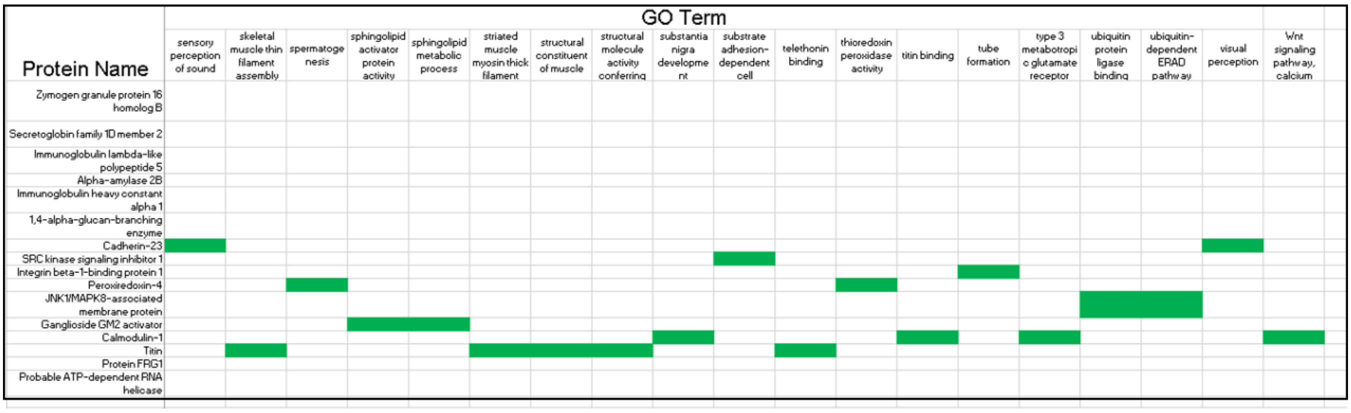

Supplementary Table 2 contd. Proteins identified from the LC-MS analysis and the molecular processes in which they are involved, as annotated on UniProt.org through phylogenetic-based propagation of functional gene ontology (GO) annotations.[2]

Protein Name	GO Term: sarcomerogenesis	sensory perception of light stimulus	sensory perception of sound	skeletal muscle thin filament assembly	spermatogenesis	sphingolipid activator protein activity	sphingolipid metabolic process	striated muscle myosin thick filament	structural constituent of muscle	structural molecule activity conferring	substantia nigra development	substrate adhesion-dependent cell	telethonin binding	thioredoxin peroxidase activity	titin binding	tube formation	type 3 metabotropic glutamate receptor	ubiquitin protein ligase binding	ubiquitin-dependent ERAD pathway	[illegible]
Zymogen granule protein 16 homolog B																				
Secretoglobin family 1D member 2																				
Immunoglobulin lambda-like polypeptide 5																				
Alpha-amylase 2B																				
Immunoglobulin heavy constant alpha 1																				
1,4-alpha-glucan-branching enzyme																				
Cadherin-23		■	■																	■
SRC kinase signaling inhibitor 1												■								
Integrin beta-1-binding protein 1																■				
Peroxiredoxin-4					■									■						
JNK1/MAPK8-associated membrane protein																		■	■	
Ganglioside GM2 activator						■	■													
Calmodulin-1											■				■		■			
Titin	■			■				■	■	■			■							
Protein FRG1																				
Probable ATP-dependent RNA helicase																				

Protein Name	GO Term: skeletal muscle thin filament assembly	spermatogenesis	sphingolipid activator protein activity	sphingolipid metabolic process	striated muscle myosin thick filament	structural constituent of muscle	structural molecule activity conferring	substantia nigra development	substrate adhesion-dependent cell	telethonin binding	thioredoxin peroxidase activity	titin binding	tube formation	type 3 metabotropic glutamate receptor	ubiquitin protein ligase binding	ubiquitin-dependent ERAD pathway	visual perception	Wnt signaling pathway, calcium
Zymogen granule protein 16 homolog B																		
Secretoglobin family 1D member 2																		
Immunoglobulin lambda-like polypeptide 5																		
Alpha-amylase 2B																		
Immunoglobulin heavy constant alpha 1																		
1,4-alpha-glucan-branching enzyme																		
Cadherin-23																	■	
SRC kinase signaling inhibitor 1									■									
Integrin beta-1-binding protein 1													■					
Peroxiredoxin-4		■									■							
JNK1/MAPK8-associated membrane protein															■	■		
Ganglioside GM2 activator			■	■														
Calmodulin-1								■				■		■				■
Titin	■				■	■	■			■								
Protein FRG1																		
Probable ATP-dependent RNA helicase																		

Supplementary Table 2 contd. Proteins identified from the LC-MS analysis and the molecular processes in which they are involved, as annotated on UniProt.org through phylogenetic-based propagation of functional gene ontology (GO) annotations.[2]

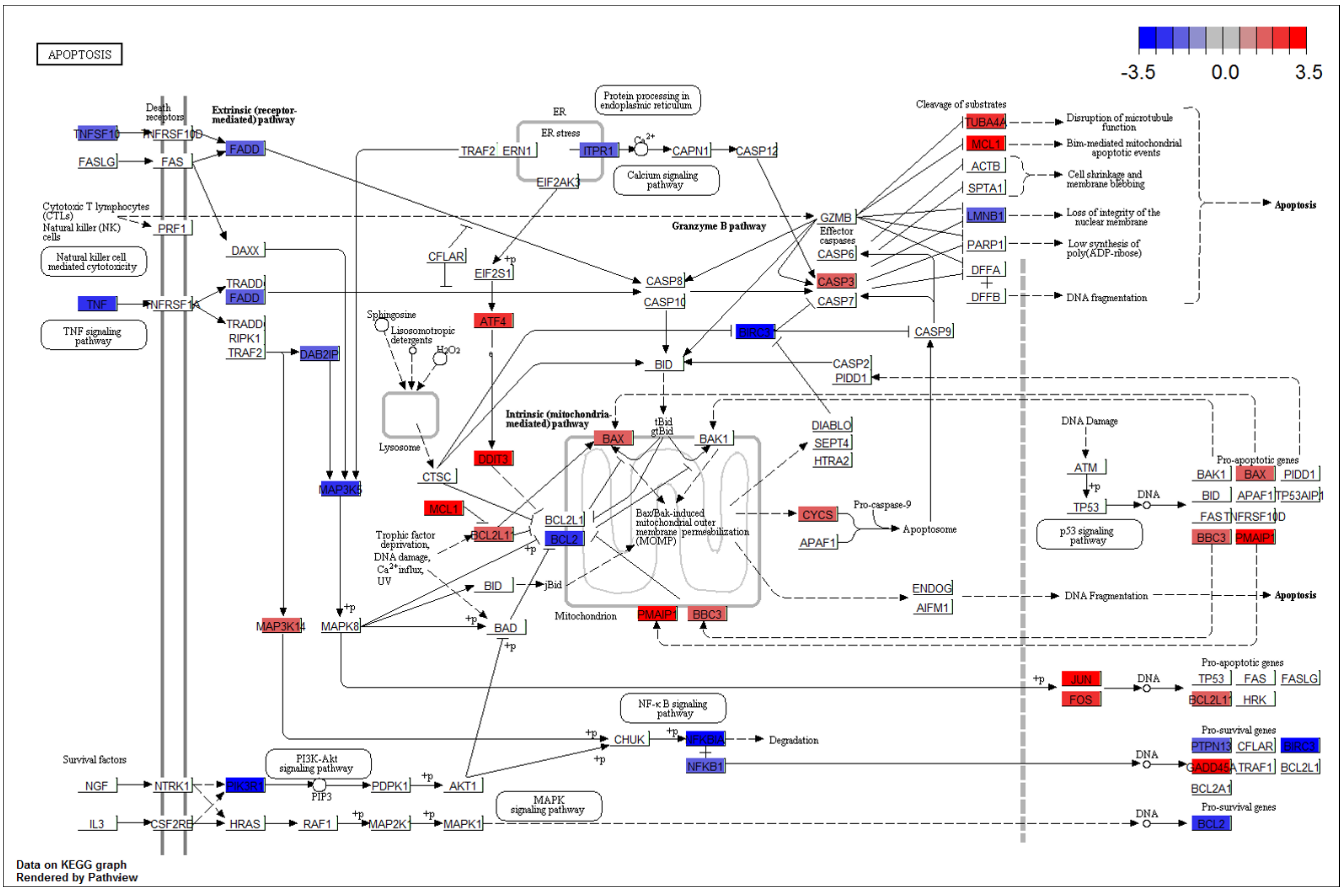

Supplementary Figure 5. Microarray based analysis for apoptosis related KEGG pathways for ionic gold treatment: Log-Fold-Change of gene regulation represented through apoptosis related KEGG pathways quantified through microarray analysis comparing Au-PEG treatments against untreated MCF-7 cells.

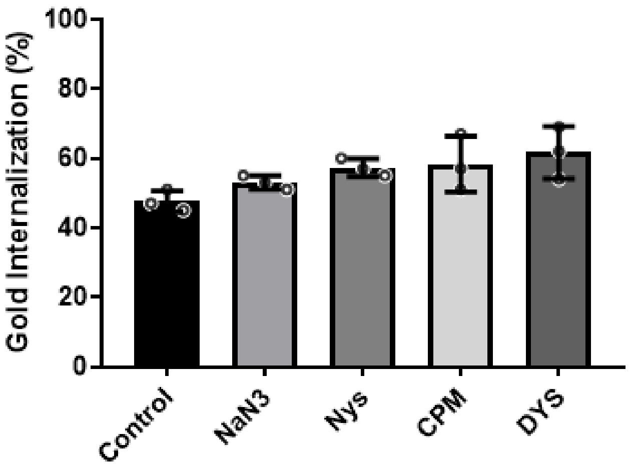

Supplementary Figure 6. Investigation of gold internalization pathway: Percentage of gold internalization in MCF-7 cells via application of ionic Au-PEG clusters at 0.24 mM Au^{3+}(control), or ionic Au-PEG clusters in presence of several endocytic inhibitors is shown. Different endocytic inhibitors employed in this study were sodium azide (NaN_3), 2-deoxyglucose, nystatin (Nys), chlorpromazine (CPM), and dynasore. Error bars are standard deviations of the mean (n=3 biologically independent samples).

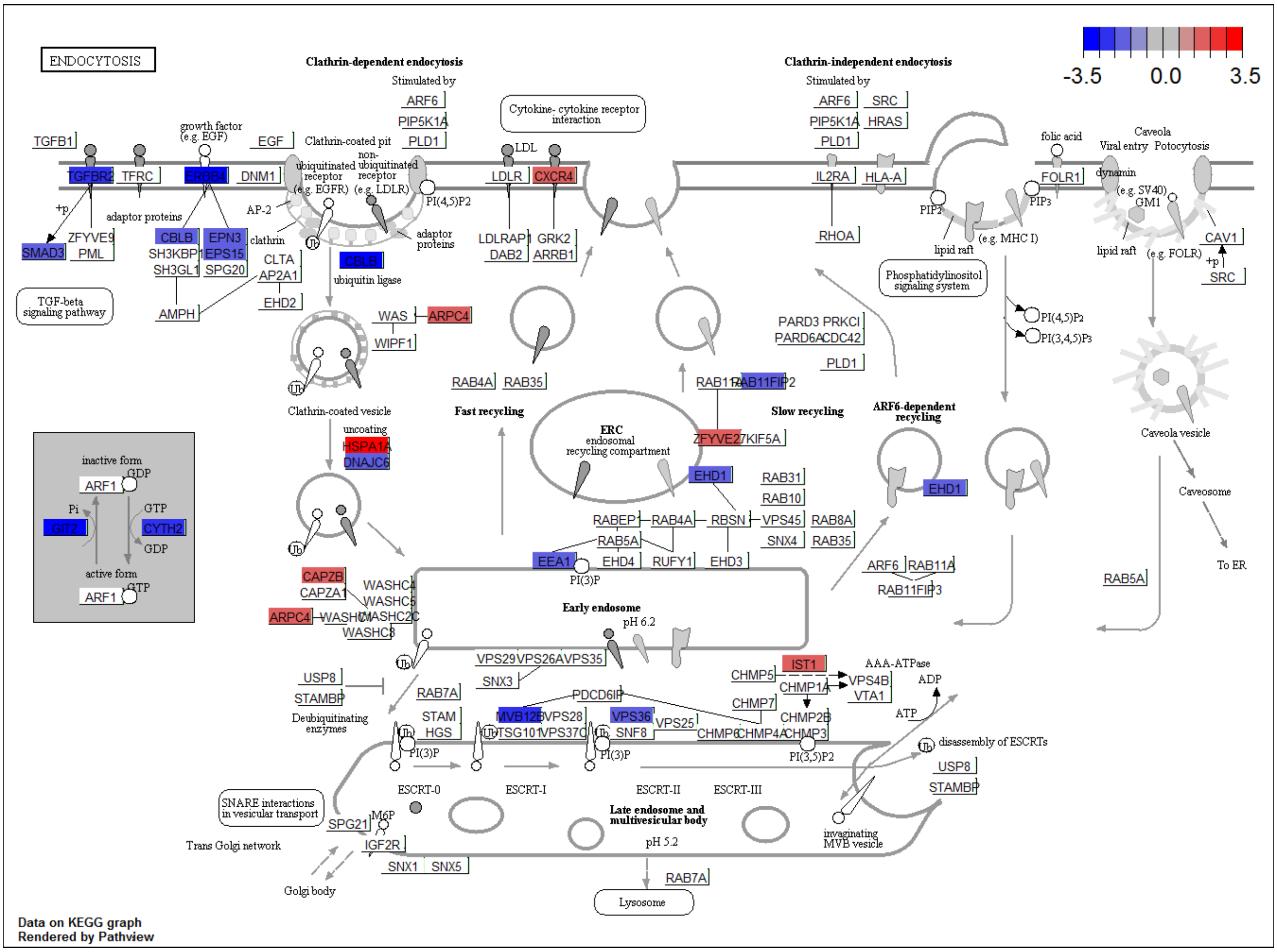

Supplementary Figure 7. Microarray based analysis for endocytosis related KEGG pathways for ionic gold treatment: Log-Fold-Change of gene regulation represented through endocytosis related KEGG pathways quantified through microarray analysis comparing Au-PEG treatments against untreated MCF-7 cells.

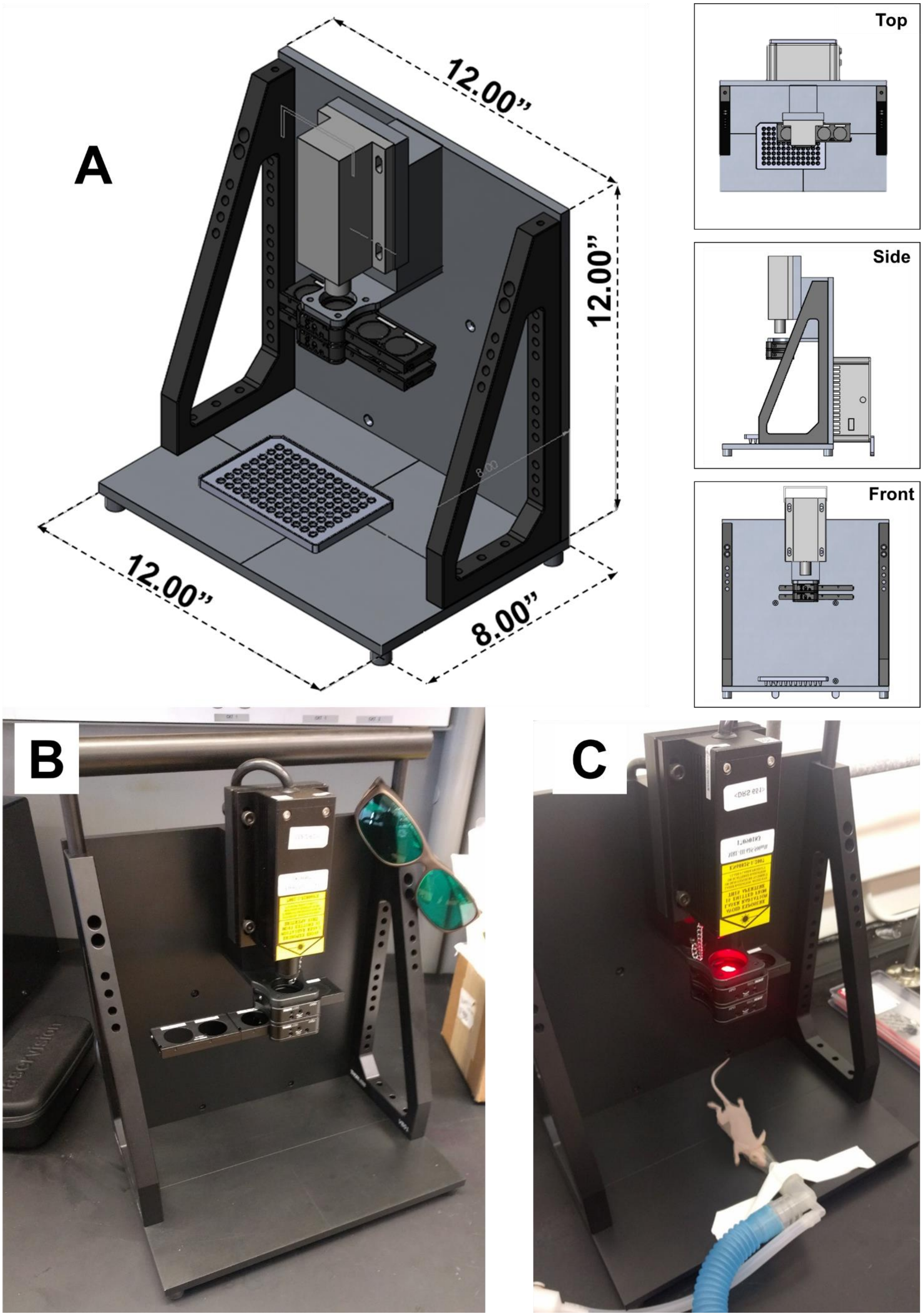
A
12.00”
12.00”
12.00”
8.00”
Top
Side
Front
B
C

Supplementary Figure 8. Schematic set up for the photothermal ablation therapy: Computer-aided design (CAD) image of laser mount for photothermal ablative therapy (to scale with RTLMRL-635-500-5 Roithner Lasertechnik cw laser), with top, side, and front views (A). Image of complete setup (B). Image of laser setup with positioned mouse (C).

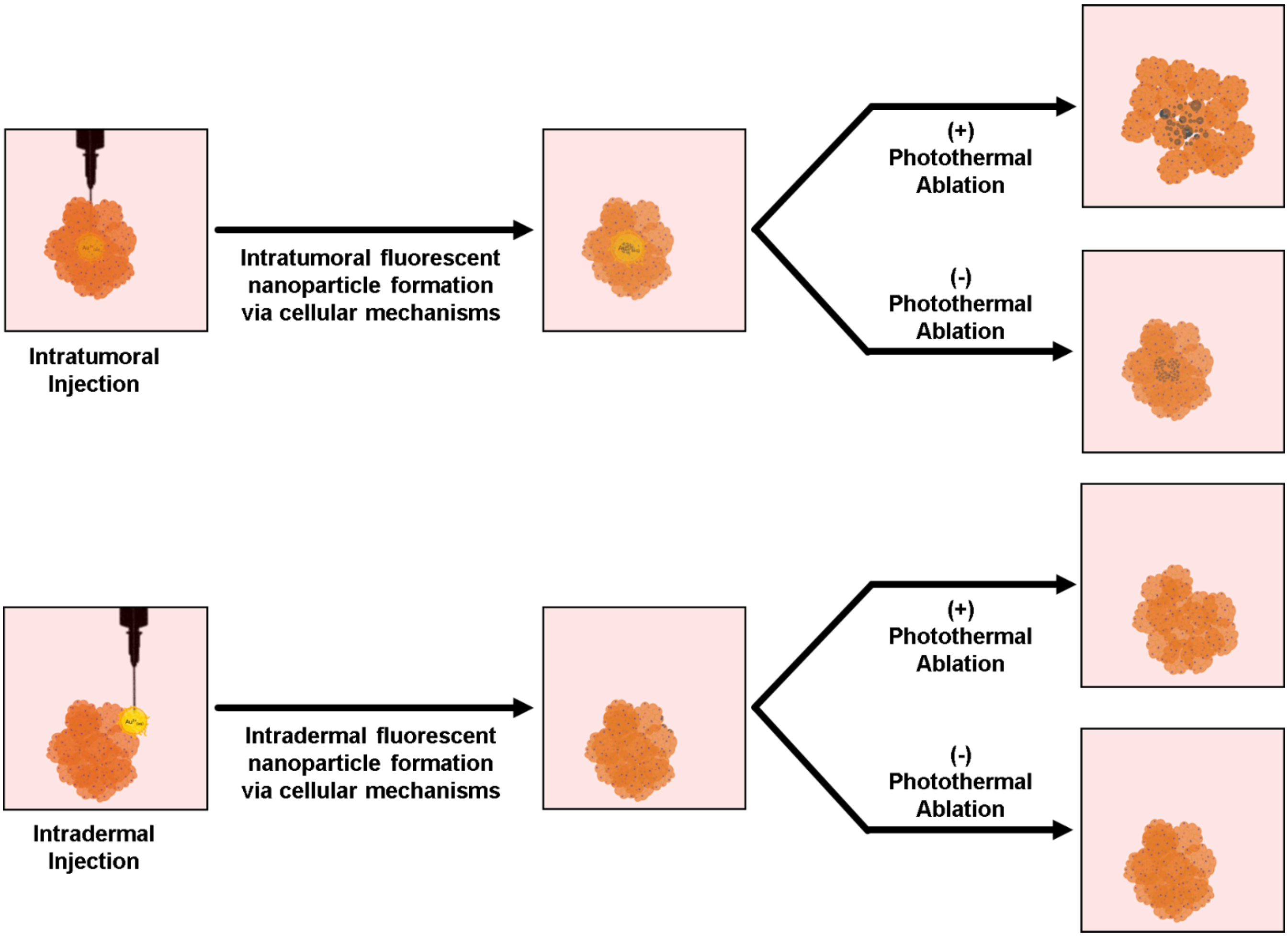

Supplementary Figure 9. Schematic diagram of nanoparticle formation: Artistic representation of how applications of ionic Au-PEG clusters will induce nanoparticle formation through successful intratumoral or intradermal applications with and without photothermal ablation.

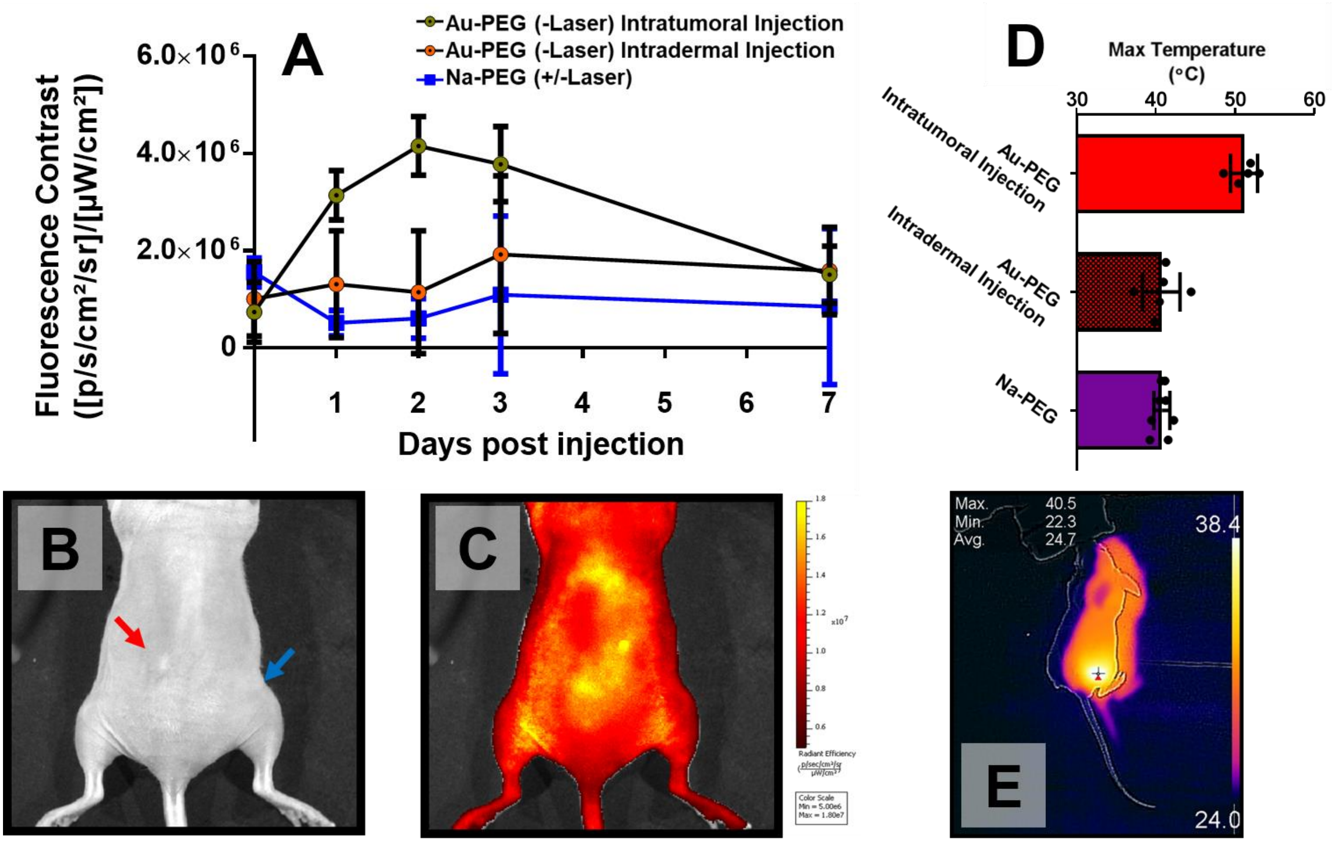

Supplementary Figure 10. *In vivo* efficacy of the intratumorally generated gold nanoparticles: (A) Fluorescence contrast afforded via IVIS imaging (430_{Ex} 840_{Em}) between on-site and off-site across one week period of treatments for Au-PEG with no photothermal treatment with intratumoral injections (Yellow), Au-PEG with intradermal injections (Orange), or Na-PEG (Blue). Error bars are standard deviation of the mean ($n \geq 3$ biologically independent animals). *The 1 day Au-PEG (-Laser) / Off site (Intradermal Injection) sample represents 2 biologically independent animals. Bright field (B) and fluorescence images (C) representitive of mice with Au-PEG with intradermal injection (three days post injection) with arrows indicating injection sites for either Na-PEG (Blue) or Au-PEG (Red). Temperature maximums averaged across laser treatments (D) as determined through FLIR IR thermal imaging with representitive thermal image of laser treatments made to mice with Au-PEG with intradermal injection (E). Error bars in for D are standard deviations of the mean ($n \geq 5$ laser treatments).

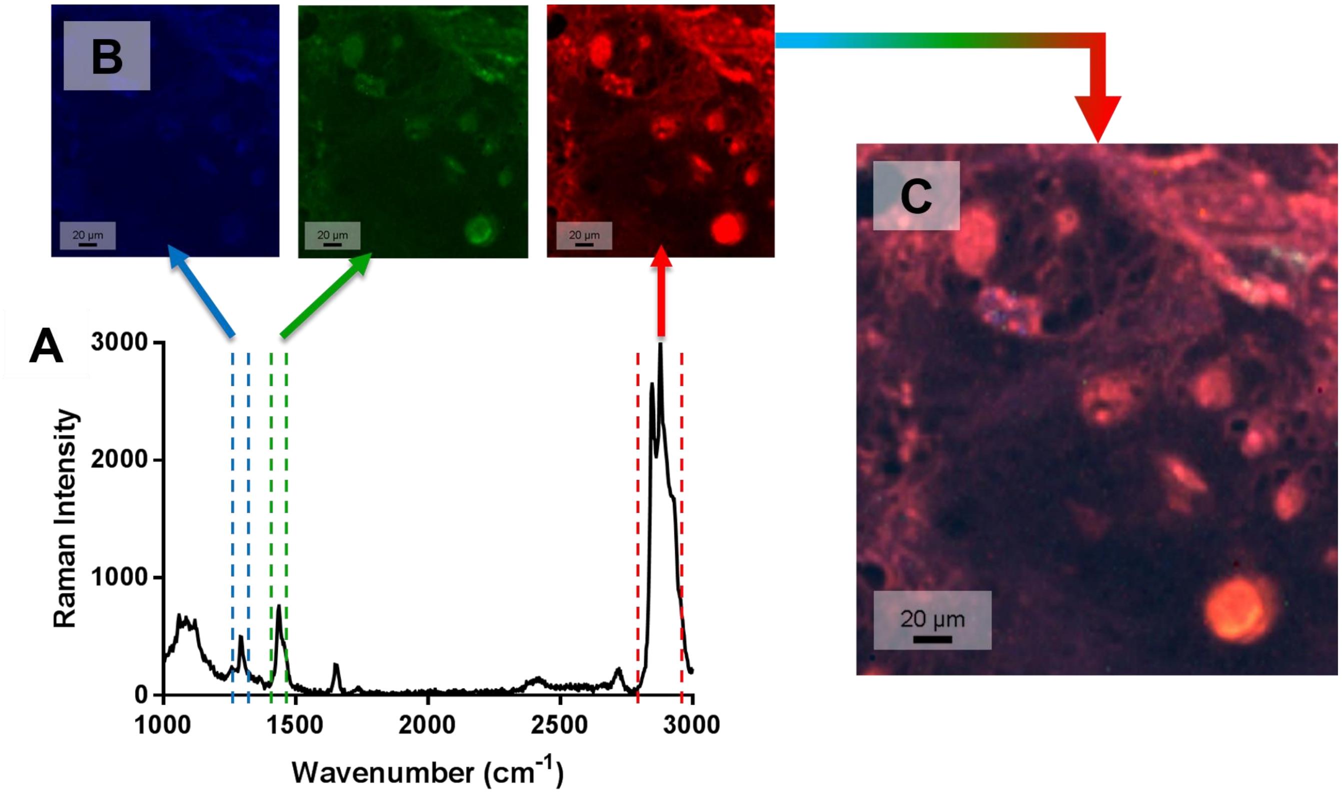

Supplementary Figure 11. Raman mapping of gold nanoparticles: Illustration of how peak intensities for regions 1250-1350, 1446-1477, and 2849-2969 cm^{-1} (A) were used for generating Blue, Green, and Red color maps (B) and were then combined for Raman mapping of unstained histological sections (C).

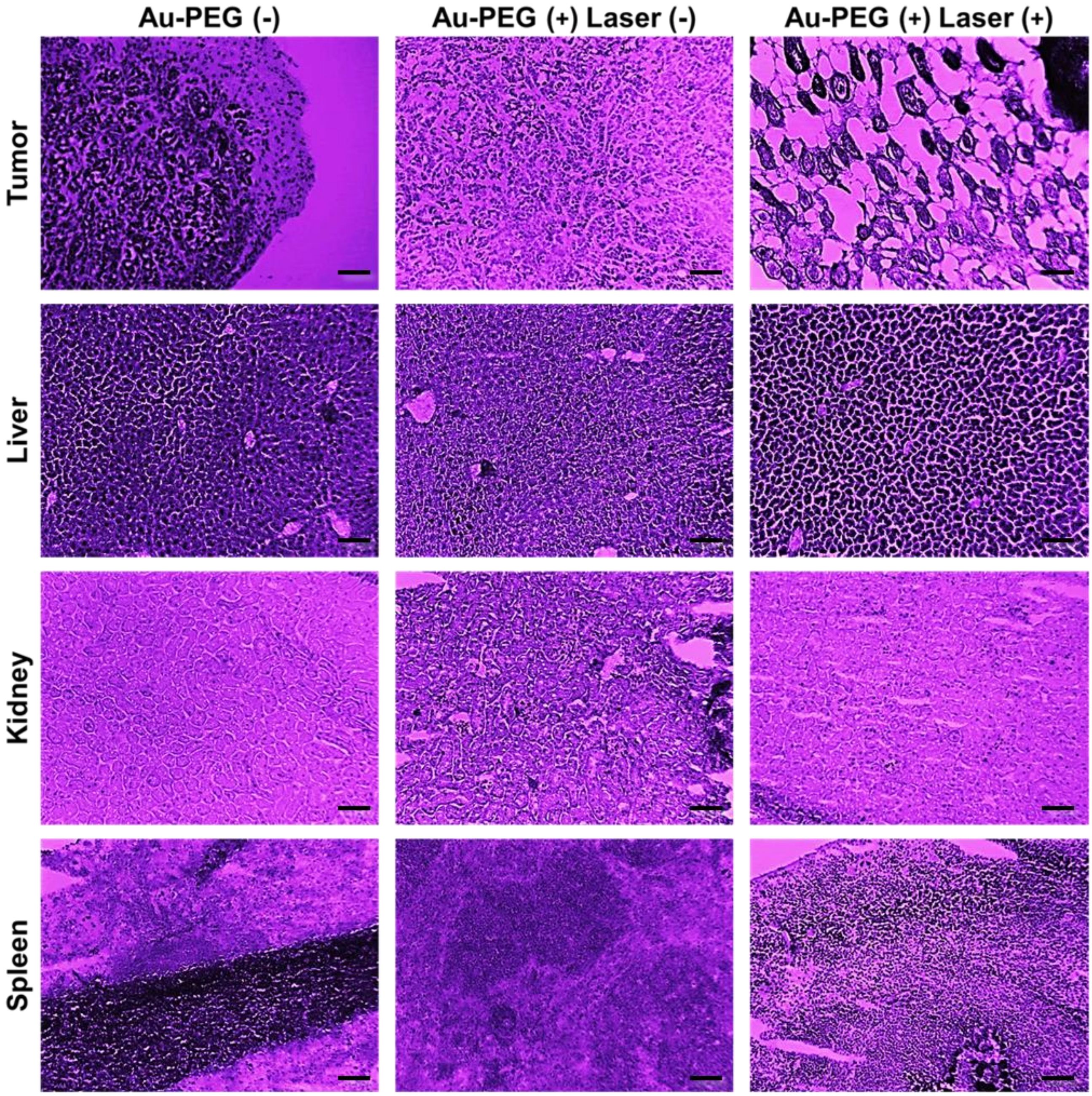

Supplementary Figure 12. Evaluating *in vivo* toxicity: Representitive H&E stained tissue sections after microtomy from animals having received Au-PEG ± and/or laser treatments ± with Au-PEG. Scale bars are 100 μm.

Supplementary References.

1. Binder, J. X. *et al.* COMPARTMENTS: unification and visualization of protein subcellular localization evidence. *Database* **2014,** bau012-bau012 (2014).
2. Gaudet, P., Livstone, M. S., Lewis, S. E. & Thomas, P. D. Phylogenetic-based propagation of functional annotations within the Gene Ontology consortium. *Brief. Bioinform.* **12,** 449–62 (2011).
