# Supplementary material for: Intratumoral generation of photothermal gold nanoparticles through a vectorized biomineralization of ionic gold
Source: Nat Commun. 2020 Sep 10;11:4530. doi: 10.1038/s41467-020-17595-6 (PMC7483505; doi:10.1038/s41467-020-17595-6)
Supplement: Supplementary file 2 — Reporting Summary [file 41467_2020_17595_MOESM2_ESM.pdf]

nature research

Corresponding author(s): Dipanjan Pan

Last updated by author(s): Jul 3, 2020

Reporting Summary

Nature Research wishes to improve the reproducibility of the work that we publish. This form provides structure for consistency and transparency in reporting. For further information on Nature Research policies, see our Editorial Policies and the Editorial Policy Checklist.

Statistics

For all statistical analyses, confirm that the following items are present in the figure legend, table legend, main text, or Methods section.

n/a	Confirmed	
☐	☒	The exact sample size (*n*) for each experimental group/condition, given as a discrete number and unit of measurement
☐	☒	A statement on whether measurements were taken from distinct samples or whether the same sample was measured repeatedly
☒	☐	The statistical test(s) used AND whether they are one- or two-sided<br>*Only common tests should be described solely by name; describe more complex techniques in the Methods section.*
☒	☐	A description of all covariates tested
☒	☐	A description of any assumptions or corrections, such as tests of normality and adjustment for multiple comparisons
☒	☐	A full description of the statistical parameters including central tendency (e.g. means) or other basic estimates (e.g. regression coefficient) AND variation (e.g. standard deviation) or associated estimates of uncertainty (e.g. confidence intervals)
☒	☐	For null hypothesis testing, the test statistic (e.g. *F*, *t*, *r*) with confidence intervals, effect sizes, degrees of freedom and *P* value noted<br>*Give P values as exact values whenever suitable.*
☒	☐	For Bayesian analysis, information on the choice of priors and Markov chain Monte Carlo settings
☐	☒	For hierarchical and complex designs, identification of the appropriate level for tests and full reporting of outcomes
☒	☐	Estimates of effect sizes (e.g. Cohen's *d*, Pearson's *r*), indicating how they were calculated

Our web collection on statistics for biologists contains articles on many of the points above.

Software and code

Policy information about availability of computer code

Data collection	The TEM images were recorded on a Gatan UltraScan 2kx2k CCD. These CCD images were processed and analyzed with ImageJ (http://rsbweb.nih.gov/ij/) version 1.48. The hyperspectral analysis software ENVI was compiled the hyperspectral and spatial data into a datacube, in which each pixel contained spectral data. Using the Spectral Angle Mapper (SAM) algorithm, the spectral libraries were then compared to their respective images.
Data analysis	The data were analyzed by GraphPad Prism, Origin, ChemDraw and microsoft powerpoint software. The flow cytometry results were analysed and plotted using Flowpy.

For manuscripts utilizing custom algorithms or software that are central to the research but not yet described in published literature, software must be made available to editors and reviewers. We strongly encourage code deposition in a community repository (e.g. GitHub). See the Nature Research guidelines for submitting code & software for further information.

Data

Policy information about availability of data

All manuscripts must include a data availability statement. This statement should provide the following information, where applicable:

- Accession codes, unique identifiers, or web links for publicly available datasets
- A list of figures that have associated raw data
- A description of any restrictions on data availability

The authors declare that the data supporting all the findings of this study are available within the article and Supporting Information files. Zip files for Microarray data can be downloaded using the following link (https://uofi.box.com/s/b10c77c0anr5q4v7sm9k313w5r8fc92z); CSV files for protein MS data can be downloaded using the following links: Top gel portion (https://uofi.box.com/s/9l6j732etrh2uqy17o0f3p8aqvp5p15a); Bottom gel portion (https://uofi.box.com/s/xjb209go4okmvba69bx405wegrv3rdmf). All the other relevant data are available from the author upon reasonable request.

Field-specific reporting

Please select the one below that is the best fit for your research. If you are not sure, read the appropriate sections before making your selection.

- [x] Life sciences
- [ ] Behavioural & social sciences
- [ ] Ecological, evolutionary & environmental sciences

For a reference copy of the document with all sections, see nature.com/documents/nr-reporting-summary-flat.pdf

Life sciences study design

All studies must disclose on these points even when the disclosure is negative.

Sample size	In Figure 5A, we have considered the standard deviation of the mean from n ≥ 3 biologically independent animals and n ≥ 5 laser treatments in Figure 5B. For Supplementary figure 5 we have considered ≥ 16 proteins, Supplementary figure 6 considered n = 3 biologically independent samples, Supplementary figure 8 undertaken n = 3 biologically independent samples, Supplementary figure 12 also shows n ≥ 3 biologically independent animals. The 1 day Au-PEG (-Laser) / Off site (Intradermal Injection) sample represents 2 biologically independent animals. Also for supplementary figure 12D, we have considered n ≥ 5 of laser treatments.
Data exclusions	No data were excluded from the analyses.
Replication	All attempts for replication of the presented data in this manuscript were successful.
Randomization	The allocation of groups were random.
Blinding	The investigators were blinded during group allocation/ analyses.

Reporting for specific materials, systems and methods

We require information from authors about some types of materials, experimental systems and methods used in many studies. Here, indicate whether each material, system or method listed is relevant to your study. If you are not sure if a list item applies to your research, read the appropriate section before selecting a response.

Materials & experimental systems

n/a	Involved in the study	
x		Antibodies
	x	Eukaryotic cell lines
x		Palaeontology and archaeology
	x	Animals and other organisms
x		Human research participants
x		Clinical data
x		Dual use research of concern

Methods

n/a	Involved in the study	
x		ChIP-seq
	x	Flow cytometry
x		MRI-based neuroimaging

Eukaryotic cell lines

Policy information about cell lines

Cell line source(s)	ATCC
Authentication	None of the cell lines used were authenticated.
Mycoplasma contamination	The cell line used were not tested for mycoplasma contamination, but cultured with proper aesthetic condition with necessary antimicrobial reagents.
Commonly misidentified lines (See ICLAC register)	The present study does not involve any cancer cell lines those are listed in the register of misidentified cell lines. MCF-7 cells (ER (+) human breast cancer cells) were sourced from ATCC (catalog no. HTB-22).

Animals and other organisms

Policy information about studies involving animals; ARRIVE guidelines recommended for reporting animal research

Laboratory animals	To investigate potential theranostic applications pre-clinically, we generated and treated MCF-7 xenograft tumors in female J/NU immunodeficient double knockout mice. Female J/Nu mice (2-3 weeks old) were bought from Jackson Laboratories, U.S.A. Upon arrival, mice were allowed 2 weeks for acclimation. Animals were group housed with free access to food and water, and individuality marked through ear punching.

Wild animals	The study did not involve wild animals.
Field-collected samples	The study did not involve samples collected from the field.
Ethics oversight	The experimental protocol received ethical approval and was approved by the Institutional Animal Care and Use Committee (IACUC), University of Illinois at Urbana–Champaign, and satisfied all University and National Institutes of Health (NIH) rules for the humane use of laboratory animals. All the animal experiments were complied by the relevant ethical regulations for animal testing and research and carried out in accordance with the approved guidelines.

Note that full information on the approval of the study protocol must also be provided in the manuscript.

Flow Cytometry

Plots

Confirm that:

- [ ] The axis labels state the marker and fluorochrome used (e.g. CD4-FITC).
- [x] The axis scales are clearly visible. Include numbers along axes only for bottom left plot of group (a 'group' is an analysis of identical markers).
- [x] All plots are contour plots with outliers or pseudocolor plots.
- [x] A numerical value for number of cells or percentage (with statistics) is provided.

Methodology

Sample preparation	MCF-7 cells (ER (+) human breast cancer cells) sourced from ATCC (catalog no. HTB-22). At the end of the experiment, animals were sacrificed via anesthesia overdose. Organ tissues (tumors, sentinel lymph nodes, liver, kidneys, and spleen) were immediately excised and frozen in cassettes containing optimal cutting temperature (OCT) compound. Embedded tissue blocks were clamped in microtome and sectioned at 6 µm thickness. Tumor and sentinel lymph node slices were either left unstained (for Raman mapping) or stained with hematoxylin and eosin (H&E) for bright field imaging.
Instrument	For Raman mapped histology, unstained tissue sections were imaged in the reflection mode via LabRAM HR 3D Horiba confocal microscope with 532 nm wavelength excitation laser with power set at 8mW. The laser light was focused through a LWD Visible 20x, NA 0.40, objective into the sample plane and scattering was recorded in the reflection geometry using a spectrograph coupled with an Andor Newton back-illuminated EMCCD camera.
Software	Samples were analyzed using a Guava EasyCyte Plus Flow cytometer. The results were analysed and plotted using Flowpy.
Cell population abundance	To further characterize the cytotoxicity of our ionic gold delivery platform, we performed flow cytometry analysis. Cells (MCF-7, 10000 cells/well) were grown in 24 well plate for 24 hours before treating with different samples prepared for this study for 4 hours. At the end of incubation, cells were trypsinized and collected in 0.2% FBS containing DPBS.
Gating strategy	For each sample, data from 5000 single cell events were collected for 3 minutes, in triplicates, and forward scatter vs side scatter information were recorded.

- [ ] Tick this box to confirm that a figure exemplifying the gating strategy is provided in the Supplementary Information.
